# Persistence of genetically engineered canola populations in the U.S. and the adventitious presence of transgenes in the environment

**DOI:** 10.1371/journal.pone.0295489

**Published:** 2024-05-22

**Authors:** Steven E. Travers, D. Bryan Bishop, Cynthia L. Sagers

**Affiliations:** 1 Department of Biological Sciences, North Dakota State University, Fargo, North Dakota, United States of America; 2 Department of Biology, Concordia College, Morehead, Minnesota, United States of America; 3 College of Integrated Sciences and Arts, Arizona State University, Tempe, Arizona, United States of America; University of Guelph, CANADA

## Abstract

Feralization of genetically engineered (GE) crops increases the risk that transgenes will become integrated into natural and naturalizing plant populations. A key assumption of the management of GE crops is that populations of escaped plants are short-lived and therefore the risks they pose are limited. However, few populations of escaped crop plants have been tracked over the long term so our understanding of their persistence in ruderal or natural landscapes is limited. We repeated a large-scale road survey of feral GE canola populations in North Dakota, USA, initially conducted in 2010. Our objectives in 2021 were to determine the current distribution of feral canola populations, and to establish the relative frequency of GE and non-GE phenotypes in populations of canola throughout North Dakota. Our results indicate that, although the incidence of feral canola was less in 2021 than 2010, escaped canola populations remain common throughout the state. The prevalence of alternate forms of GE herbicide resistance changed between surveys, and we found an overabundance of non-GE plants compared to the frequency of non-transgenic forms in cultivation. Indirect evidence of persistence includes sampling plants with multiple transgenic traits, and finding populations far from transportation routes. We conclude that feral canola populations expressing transgenic herbicide resistance are established outside of cultivation, that they may be under selection for loss of the transgene, but that they nonetheless pose long-term risks by harboring transgenes in the unmanaged landscape.

## Introduction

The adventitious presence of transgenes in production systems continues to pose significant risks to U.S. agriculture. Detection of transgenes in foodstuffs has disrupted international and domestic markets, stalled supply chains, and created mistrust among consumers [1]. Durisin and Wilson [2] estimated that the threat of contamination of conventional and organic crops by genetically engineered (GE) varieties cost producers $6.3B US when food companies and foreign markets rejected transgene-contaminated supplies. Limiting the movement of transgenes in agricultural systems is central to reducing market losses, but such efforts are frustrated by the biology of plants and their ability to out-maneuver containment strategies. Numerous crop species have escaped cultivation and established persistent populations outside of cultivation [3]. Outside of managed croplands, escaped crops may evolve rapidly to become more closely adapted to, and tolerant of the non-agronomic environment [4]. Once established, they constitute a gene pool capable of generating weedy pests that carry the additional risks of harboring beneficial transgenes [5]. The risks of an adventitious presence, then, are enhanced when GE crop species escape cultivation to form stable, naturalized populations in the unmanaged landscape.

De-domestication, or feralization, of GE crops increases the likelihood that transgenes will escape cultivation and integrate with wild populations of closely related plants. De-domestication is an evolutionary process by which domesticated plants or animals escape intensive management by humans to form independent reproducing populations [3]. De-domesticates are known to originate from within crop species through multiple routes: through mutation and selection (endo-ferals), by crossing between distinct populations or land races (exo-endo ferals), or by hybridizing with wild relatives (exoferals) [3]. Once established, the genetic architecture of de-domesticated populations may be further shaped by hybridization and introgression among wild, feral, and domesticated forms [6–8]. Wild traits, such as seed-shattering, asynchronous flowering and seed dormancy, are at times quickly recovered by de-domesticates as a product of selection in non-agronomic habitats [4]. The resulting feral populations, now more closely adapted to local environments and tolerant of competition, are rich targets for ongoing gene flow from agricultural fields [9]. When they compete with related crops, or introduce deleterious traits to commercial fields, de-domesticates pose a threat to the integrity and profitability of commercial production systems.

Among domesticated species, kohl crops have an evolutionary history punctuated with repeated bouts of feralization, hybridization and introgression [8, 10, 11]. Canola (*Brassica napus* L.) (2n = 4n = AACC) is a hybrid of *B*. *rapa* (2n = 20, AA) and *B*. *oleracea* (2n = 18, CC) [12]. It has become one of the most important oilseed crops worldwide and has been cultivated extensively for more than 100 years [13]. It is thought to have been domesticated recently (within the last 300–400 years) [14]. Populations of domesticated canola growing outside of cultivation have been reported from Belgium, Austria, Denmark, France, Germany, U.K., Australia, the Netherlands, and New Zealand [1]. “Wild” traits still expressed in commercial canola, such as seed shattering, may contribute to its ready escape from cultivation [1]. Moreover, canola seeds retain partial dormancy and may remain viable in the soil seedbank for up to three years [15, 16]. The combined effects of seed loss on harvest and seed dormancy rapidly stock the soil seed bank, contributing to the high incidence of volunteer canola in and around agricultural fields [17, 18].

The U.S. was an early adopter of GE canola in the mid-1990s and now nearly all U.S. canola is GE [19]. In 2011, we described the presence of escaped GE herbicide resistant (GE HR) canola populations North Dakota, USA, where most of the U.S. canola is grown. More than 75% of the plants sampled from roadside populations were GE HR and most populations contained a mixture of GE HR phenotypes, either glyphosate resistance or glufosinate resistance [20]. (Both GE HR and non-GE plants are referred to here as feral.) The source of these populations has yet to be determined, but it was generally believed they result from seed spill during transportation [21]. We repeated the survey in 2021 with the objectives to:

- Determine the current distribution of feral canola populations in North Dakota, USA
- Establish the relative frequency of GE HR traits in North Dakota populations
- Investigate the distribution and frequency of non-GE plants in roadside samples

Between census periods, U.S. production of canola increased, with a reported increase in the cultivation of glufosinate resistant varieties. Therefore, if seed spill is the dominant factor maintaining feral canola populations, as has been argued [21, 22], we anticipate an increase in the number and size of feral populations, and a change in the phenotypic profile of feral populations.

## Methods

### Study design

In 2021, we conducted systematic roadside surveys to quantify the presence and abundance of feral GE HR and non-GE canola populations in North Dakota, USA, beginning 14 June 2021 and continuing through 7 July 2021. No permits or approvals were required for this work as all samples were collected in the public right of way and no protected species were sampled. In this study, as in the earlier one, our goals was to characterize the distribution and abundance of transgenic canola across a wide area throughout the region where canola is grown. Given the constraints of time and resources, we chose to optimize area sampled over depth of sampling at each site.

### Sampling

We adopted methods similar to the survey of 2010 [20]. Sampling was conducted early in the summer prior to the onset of flowering of cultivated canola. Field crews established transects on major east-west highways throughout the state. A 2×50 m quadrat was established every 8.05 km (5 miles) of roadway on one or both sides of the road, where traffic permitted, by walking 50 m along the road margin and observing one meter to the right and left. Inside that sampling area, all identifiable *Brassica napus* plants were counted. *Brassica napus* is distinguishable from other *Brassica* species by its glabrous leaves with a rubbery texture and clasping bases [23]. When canola was present at a sampling site, one randomly selected plant was collected, photographed, tested for transgene expression, and archived as a voucher specimen. We drove a total of 6373 km and sampled 62.5 km of roadside habitat (1.0% of the distance driven).

### Detection

Leaf fragments from voucher specimens were tested for the presence of CP4 EPSPS protein (confers tolerance to glyphosate herbicide) and PAT protein (confers tolerance to glufosinate herbicide) with TraitChek^™^ immunological lateral flow test strips (# 10001326, 10001313, Romer Labs, Inc., Newark, DE). Previous studies have demonstrated the utility of the lateral flow strips in detecting the expression of transgenes from field collected samples [20, 24, 25]. Test strips are not available for a third, non-GE resistance trait, resistance to Clearfield^™^ herbicide, which is reported to comprise less than 5% of the GE HR canola grown in the region (R Beneda, pers comm). At random intervals, single plants were tested with multiple test strips to assure that results were repeatable and reliable (CP4 EPSPS+, *N* = 9; PAT+, *N* = 15). No errors were detected during the course of the study.

### Additional surveys

To confirm a reported increase in PAT+ cultivation in 2021, we collected canola growing immediately outside of 58 agricultural fields and presumably established by seed spill. Each plant was tested for the expression of GE HR. The 58 fields were distributed evenly throughout the state and represent all of the flowering canola in cultivation that we observed during the survey period.

In 2022 we sought to determine if populations of escaped canola are composed of multiple herbicide-resistant phenotypes, evidence of multiple founder events. At nine randomly selected, large, feral canola populations, we collected 25 plants and tested for the presence of CP4 EPSPS or PAT proteins. These nine populations were chosen on the basis of having more than 100 individuals and being evenly distributed across our sampling region. In contrast to our transect sampling, study plants separated by more than 0.25m were collected haphazardly in each population. We used a chi-square contingency statistic to test the null hypothesis that the frequency of the four transgenic phenotypes (CP4 EPSPS+; PAT+; CP4 EPSPS+/PAT+; non-GE) did not change between sampling years.

## Results

### 2021

**Fig 1:**
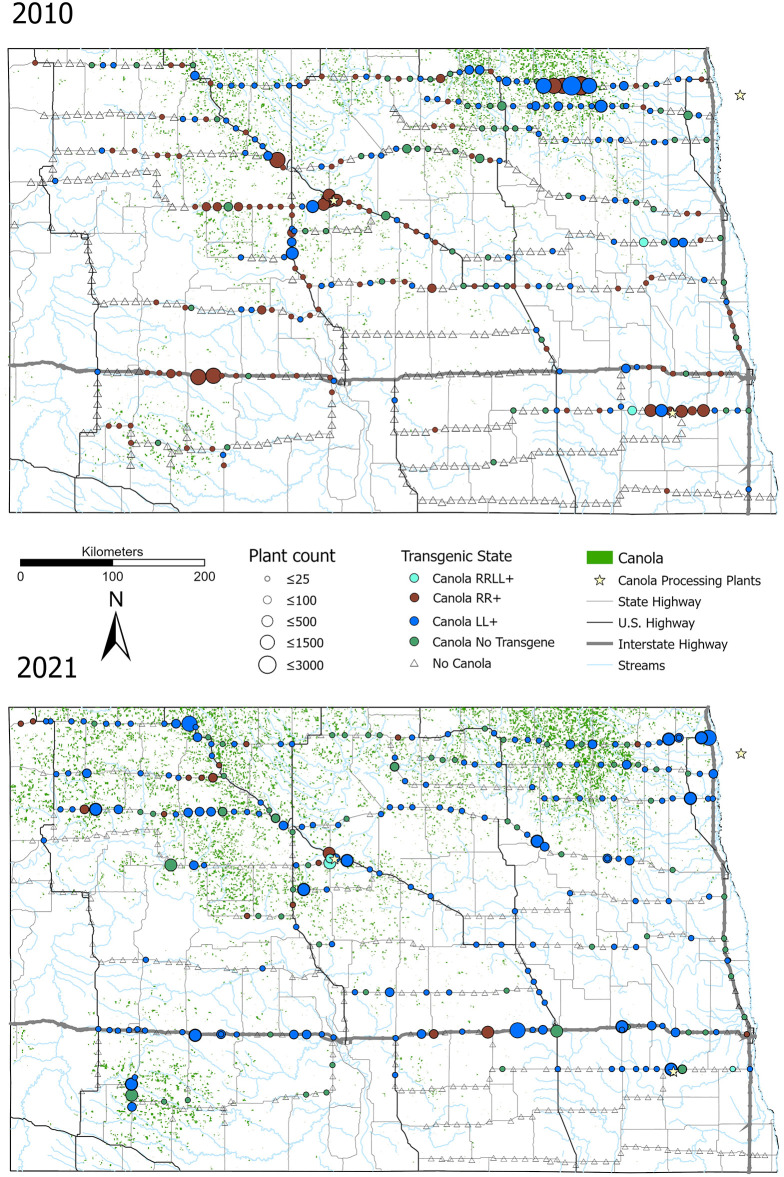
Results of 2010 and 2021 field surveys of feral canola populations in North Dakota. Solid black lines indicate major highways. Collection sites indicated by symbols: triangles = no canola; circles = canola present. Shading indicates transgenic character state: blue = PAT+ (LL); red = CP4 EPSPS+ (RR); turquoise = PAT+/CP4 EPSPS+ (RR/LL); green = non-GE. Circle diameter indicates estimate of local population size. Green stippling indicates locations of canola production fields in 2009 (panel A) and 2020 (panel B). Oilseed processing plants are noted by stars. 2011 survey data from Schafer et al. 2011 [20]. Sources for GIS data layers available in S1 Appendix.

Populations of escaped canola were once again widespread throughout the state (Fig 1). In 2021, canola was present at 42.0% of the 623 road survey sampling sites. Of those, 76.0% expressed at least one transgene: 67.0% were positive for PAT (glufosinate resistance); 8.0% were positive for CP4 EPSPS (glyphosate resistance); and 1.1% (3/262) expressed both forms of GE HR (S1 Table). Densities of canola plants at collection sites ranged from 0 to 10 plants m^−2^ with an average of 0.4 plants m^−2^.

Sampling within large populations of feral canola revealed a mix of both GE HR and non-GE phenotypes. In 2022, the nine large, feral populations sampled extensively were comprised of a mix of resistance phenotypes (CP4 EPSPS+, PAT +, CP4 EPSPS+/PAT+, non-GE).

All cultivated canola sampled in 2021 expressed GE HR. Of the specimens collected outside of agricultural fields, 89.7% expressed PAT; 98.6% expressed CP4 EPSPS; and 1.7% expressed both transgenes. We found evidence of neither non-GE nor Clearfield^™^ canola cultivation in our sample (both would have registered negative with the test strips available to us).

### 2021 vs. 2010

**Fig 2:**
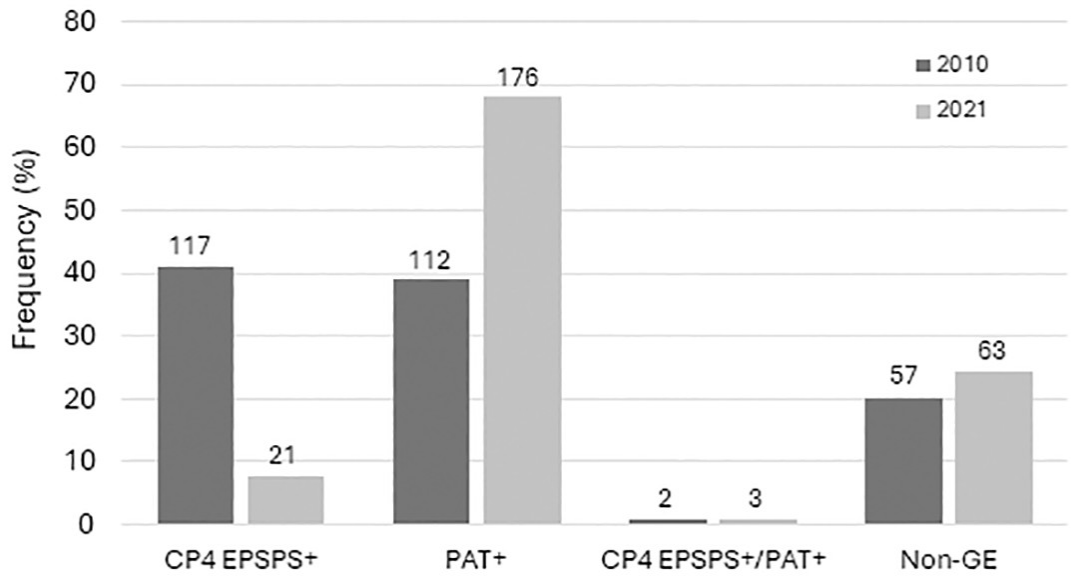
Frequency of GE and non-GE samples in 2010 and 2021 field surveys of feral canola in North Dakota. Open bars: 2010 survey; closed bars: 2021 survey. CP4 EPSPS+—glyphosate resistant; PAT+—glufosinate resistant. Numbers above bars indicate sample size. Differences between census periods in the relative frequencies of resistance phenotypes are significant (p < .001).

In 2021, we sampled a total of 623 roadside sites, 1.0% fewer than in 2010. Overall, we encountered far fewer plants in the survey in 2021 than in 2010 (9884 vs. 18,960, respectively), despite a substantial increase (approx. 26%) in the total number of acres of cultivated canola [26]. The proportion of sampling sites with canola dropped in 2021 to 42.0% from 45.0%. We detected shifts in the relative frequency of GE HR phenotypes with PAT becoming the dominant form of HR (67.0% in 2021 versus 49.0% in 2010), CP4 EPSPS becoming less frequent (10.0% in 2021 versus 51.0% in 2010), and non-GE phenotypes increasing slightly to 24.2% from 19.9% (Fig 2). Overall, the changes between census periods in the relative frequency of GE HR and non-GE phenotypes in feral populations was significant (χ^2^ = 80.5, df = 3, *N* = 551, p <0.001).

In both census periods, populations of GE HR canola were most dense along major transport routes, at construction sites and in regions of intense canola cultivation (Fig 1). On a local scale, feral populations appeared denser at junctions between major roadways, access points to crop fields and bridges, and at intersections of roadways with railway crossings. At these sites, seed spill during transport appears the most likely mechanism for the escape of GE HR canola. Nonetheless, feral canola plants seen at remote locations far from canola production, transportation, or processing facilities were encountered more frequently in 2021 (*N* = 10) than in 2010 (*N* = 3). In the second survey, in addition to growing in road verges and construction sites, we discovered feral canola in riparian habitats and natural areas, including two populations in protected areas (Sheyenne National Grassland, Upper Souris National Wildlife Refuge). Moreover, canola plants growing at streamside locations were robust and phenotypically more similar to crop canola than feral plants in road verges and construction fill.

## Discussion

### Long term trends in feral canola populations in North Dakota

Feral canola remains widespread along the roadsides of North Dakota. The total number of feral canola plants in the sample, and the frequency of populations fell between the 2010 and 2021 sampling periods. The incidence of GE HR populations growing outside of cultivation decreased, despite a substantial increase in the number of cultivated acres (approx. 26%). A drop in incidence despite an increase in cultivated acres may be explained by severe drought conditions that persisted through the growing season of 2021; rainfall recorded during May through August 2021 in the centrally located city of Minot, ND, was approximately 38% below average [27].

The make-up of feral populations changed over the course of a decade. Whereas feral plants in 2010 were equally likely to be resistant to glyphosate (CP4 EPSPS+) as glufosinate (PAT+), by 2021 glufosinate resistance was nine times more common. We would expect to see this rate of change if cultivation had shifted strongly to PAT+ from CP4 EPSPS, escaped populations are maintained through seed spill and feral populations are ephemeral. Although the U.S. Department of Agriculture (USDA) no longer publishes data on GE HR canola acreages, a limited survey of agricultural fields in 2021 showed a prevalence of PAT+ cultivation. Further, conversation with a representative from a regional growers association revealed PAT+ to be more popular than CP4 EPSPS+ in recent years (B. Coleman, pers. comm.). This may be the result of an increasing number of glyphosate-resistant weeds that have evolved in the intervening decade. Currently in the U.S., 17 weed species are resistant to glyphosate whereas only three species are resistant to glufosinate [28]. In addition, large populations of feral canola express a mixture of resistance phenotypes, consistent with repeated seed spill events. Crawley et al. [22] first documented that heavy seed spill, despite high mortality rates, can maintain escaped canola populations along transit routes. Together, these findings support the idea that escaped populations are dynamic and persist under selection, and with repeated re-introduction of new seeds each growing season. By sampling along transportation routes, we undoubtedly created bias toward detecting the effects of seed spill. Future studies should sample further afield and include riparian areas and grasslands.

### Long-term persistence of feral canola

Movement by transport may explain the current distribution of feral GE HR canola along transportation routes in North Dakota, but it does not explain the incidence of feral non-GE plants. From 2010 to 2021, the frequency of non-GE canola escaped from cultivation increased to 24.2% from 19.9%, despite the near universal cultivation of GE HR canola in North Dakota. The most recent published estimate of GE HR canola is approximately 95% of cultivated acres [19]. In our small sample of canola fields in 2021, none were non-GE, however. Whether non-GE plants are recent escapes that have lost a transgene, or descended from older feral lineages [29] is uncertain. Future studies of the genetic structure of escaped populations could easily resolve questions of their origins, but progress to this end has been slowed by the complexities of genomics analysis of the *B*. *napus* tetraploid genome. Nevertheless, a handful of studies have made headway untangling the genomics and structure of feral *B*. *napus* populations [30–32] and an answer is within reach. Whether they are recent or more historical, the high incidence of non-GE forms argues for the longer-term persistence of feral *B*. *napus* in the environment and suggests that the dynamics of feral GE HR canola are more complex than initially described [33].

## Conclusions

These findings raise compelling questions about the dynamics of feral GE HR canola populations. A decadal change in transgenic phenotype frequencies in roadside feral populations may simply reflect a change in the types of GE HR canola crops planted over this time [34]. Far more compelling is the high frequency of non-GE forms in the unmanaged landscape relative to non-GE planted acreages. These plants present a rare opportunity to understand the histories of populations in the process of de-domestication and the mechanisms of transgene persistence in them. They may be recent introductions, or they may be historical populations that have persisted in the landscape before the introduction of GE HR or commercial canola. As such, they merit further analyses at the genomic and population level. Fully understanding the risks posed by the adventitious presence of transgenes will require further genomics studies of populations far away from transportation routes in areas and in areas such as North Dakota where GE HR canola production is widespread [32].

There is now evidence for self-sustaining feral canola populations in 14 countries on five continents (reviewed in [1]). In Europe and Japan, plants in feral populations are similar genetically to varieties planted in fields nearby but nonetheless show a degree of divergence [30–33]. These studies show that feral populations have higher levels of genetic diversity, and unique alleles and combinations of alleles, suggesting the admixture of wild, cultivated and feral genomes, multiple incidences of escape, and long-term persistence [30, 31]. Genetically engineered canola is reported growing outside of cultivation in six countries: USA [20], Japan [32, 34], Canada [35, 36], Switzerland [37], Argentina [38], and the Netherlands [39], yet the detailed population-level analyses required to assess their risks are few and restricted to regions where commercial GE HR canola production is limited. The structure and dynamics of these populations and the risks they pose to commercial agriculture merit further study.

## Supporting information

S1 Appendix (DOCX)

S1 Table Data collected from road surveys of GE canola in North Dakota, 2010 and 2021. Year is sampling year; sample ID is the sequential number given to each site sampled; Lat/Long is the latitude and longitude of each sampling site; transgene state is the phenotype of the canola plant, if encountered, at each site (none = no canola, null = non-GE canola, RR+ = CP4 EPSPS+ canola; LL+ = PAT+ canola: LL+RR+ = canola expressing both transgenes); collector names the project staff member responsible for the collection; Plants/m2 is the estimated density of sampled canola population.
(DOCX)
