# Supplementary material for: Persistence of genetically engineered canola populations in the U.S. and the adventitious presence of transgenes in the environment
Source: PLoS One. 2024 May 22;19(5):e0295489. doi: 10.1371/journal.pone.0295489 (PMC11111013; doi:10.1371/journal.pone.0295489)
Supplement: S1 Appendix — (DOCX) [file pone.0295489.s001.docx]

**Appendix I. GIS data sources**

ND Department of Transportation. State and Federal Roads (2021) [downloaded file].. URL: <https://gishubdata-ndgov.hub.arcgis.com/>. Accessed October 9, 2021.

US Geological Survey Streams and Rivers 2000k (2020) [downloaded file]. US Geological Survey - original linework development of the National Atlas, ND State Water Commission - final compilation, merging, attribution. URL: <https://gishubdata-ndgov.hub.arcgis.com/>. Accessed October 9, 2021.

USDA National Agricultural Statistics Service Cropland Data Layer. {2020, 2010}. Published crop-specific data layer [Online]. [downloaded file]. URL: <https://nassgeodata.gmu.edu/CropScape/>. Accessed October 9, 2021. USDA-NASS, Washington, DC.

USDA North Dakota State Boundary. Feature Service Feature Class. <https://gis.apfo.usda.gov/arcgis/rest/services/BASE/State/MapServer>

For the software used to make the map:

ESRI 2023. ArcGIS Pro^®^ ver. 3.1. Redlands, CA: Environmental Systems Research Institute. <https://www.esri.com/>
