# Supplementary material for: Persistence of genetically engineered canola populations in the U.S. and the adventitious presence of transgenes in the environment
Source: PLoS One. 2024 May 22;19(5):e0295489. doi: 10.1371/journal.pone.0295489 (PMC11111013; doi:10.1371/journal.pone.0295489)
Supplement: S1 Table — Year is sampling year; sample ID is the sequential number given to each site sampled; Lat/Long is the latitude and longitude of each sampling site; transgene state is the phenotype of the canola plant, if encountered, at each site (none = no canola, null = non-GE canola, RR+ = CP4 EPSPS+ canola; LL+ = PAT+ canola: LL+RR+ = canola expressing both transgenes); collector names the project staff member responsible for the collection; Plants/m2 is the estimated density of sampled canola population. (DOCX) [file pone.0295489.s002.docx]

Supplemental Table 1. Dataset collected from GM Canola surveys in 2010 and 2021. Year is the year a site was sampled; sample ID indicates the sequential number given to each site sampled; Lat/Long is the location of each site in North Dakota; transgene state is the genotype of the plant sampled at the site (none=no canola, null = no transgene in sampled plant, RR+ means the plant was positive for CP4 EPSPS; LL+ means it contains PAT protein; LL+RR+ is a stacked plant with both transgenes); collector is the person who collected the sample; Plants/m2 is the number of plants at the site divided by 100 square meters.

| Year | Sample ID | Latitude | Longitude | transgene state | collector | plants/m2 |
| --- | --- | --- | --- | --- | --- | --- |
| 2021 | 1 | 46.05138 | -96.7591 | none | CS | 0 |
| 2021 | 2 | 46.05138 | -96.685 | none | CS | 0 |
| 2021 | 3 | 46.05167 | -96.8044 | none | CS | 0 |
| 2021 | 4 | 46.06623 | -96.9079 | none | CS | 0 |
| 2021 | 5 | 46.06598 | -97.0104 | none | CS | 0 |
| 2021 | 6 | 46.0651 | -97.1153 | none | CS | 0 |
| 2021 | 7 | 46.07875 | -97.2188 | none | CS | 0 |
| 2021 | 8 | 46.07967 | -97.3221 | none | CS | 0 |
| 2021 | 9 | 46.07977 | -97.4261 | none | CS | 0 |
| 2021 | 10 | 46.08068 | -97.5354 | none | CS | 0 |
| 2021 | 11 | 46.08637 | -97.6335 | none | CS | 0 |
| 2021 | 12 | 46.10267 | -97.7271 | none | CS | 0 |
| 2021 | 13 | 46.10267 | -97.8412 | none | CS | 0 |
| 2021 | 14 | 46.10985 | -97.9456 | none | CS | 0 |
| 2021 | 15 | 46.10987 | -98.0492 | none | CS | 0 |
| 2021 | 16 | 46.06635 | -98.0912 | none | CS | 0 |
| 2021 | 17 | 46.00858 | -98.1306 | none | CS | 0 |
| 2021 | 18 | 46.00885 | -98.2358 | none | CS | 0 |
| 2021 | 19 | 46.0087 | -98.3499 | none | CS | 0 |
| 2021 | 20 | 46.00925 | -98.4549 | none | CS | 0 |
| 2021 | 21 | 45.98578 | -98.5304 | none | CS | 0 |
| 2021 | 22 | 45.91095 | -98.5189 | none | CS | 0 |
| 2021 | 23 | 45.83632 | -98.5177 | none | CS | 0 |
| 2021 | 24 | 46.00343 | -98.6362 | none | CS | 0 |
| 2021 | 25 | 46.0236 | -98.7142 | none | CS | 0 |
| 2021 | 26 | 46.02568 | -98.8184 | none | CS | 0 |
| 2021 | 27 | 46.02645 | -98.9227 | none | CS | 0 |
| 2021 | 28 | 46.02683 | -99.0256 | none | CS | 0 |
| 2021 | 29 | 46.02713 | -99.1295 | none | CS | 0 |
| 2021 | 30 | 46.02702 | -99.2334 | none | CS | 0 |
| 2021 | 31 | 46.03337 | -99.334 | none | CS | 0 |
| 2021 | 32 | 46.79633 | -99.3423 | none | CS | 0 |
| 2021 | 33 | 46.01778 | -99.5458 | none | CS | 0 |
| 2021 | 34 | 46.0283 | -99.6497 | none | CS | 0 |
| 2021 | 35 | 46.02538 | -99.7538 | none | CS | 0 |
| 2021 | 36 | 46.02605 | -99.8371 | none | CS | 0 |
| 2021 | 37 | 46.02877 | -99.9421 | none | CS | 0 |
| 2021 | 38 | 46.02913 | -10.0463 | none | CS | 0 |
| 2021 | 39 | 46.0873 | -10.0944 | none | CS | 0 |
| 2021 | 40 | 46.13577 | -10.1701 | none | CS | 0 |
| 2021 | 41 | 46.20355 | -10.2059 | none | CS | 0 |
| 2021 | 42 | 46.25408 | -10.2118 | none | CS | 0 |
| 2021 | 43 | 46.24657 | -10.1081 | none | CS | 0 |
| 2021 | 44 | 46.2462 | -99.989 | none | CS | 0 |
| 2021 | 45 | 46.26038 | -99.8967 | none | CS | 0 |
| 2021 | 46 | 46.26138 | -99.7903 | none | CS | 0 |
| 2021 | 47 | 46.25952 | -99.6913 | none | CS | 0 |
| 2021 | 48 | 46.25908 | -99.5833 | none | CS | 0 |
| 2021 | 49 | 46.25885 | -99.4828 | none | CS | 0 |
| 2021 | 50 | 46.27852 | -99.3732 | none | CS | 0 |
| 2021 | 52 | 46.28312 | -99.165 | none | CS | 0 |
| 2021 | 53 | 46.31188 | -99.0645 | none | CS | 0 |
| 2021 | 54 | 46.31195 | -98.955 | none | CS | 0 |
| 2021 | 55 | 46.35532 | -98.9072 | none | CS | 0 |
| 2021 | 56 | 46.35522 | -98.7977 | none | CS | 0 |
| 2021 | 57 | 46.3552 | -98.6829 | none | CS | 0 |
| 2021 | 58 | 46.35528 | -98.5781 | none | CS | 0 |
| 2021 | 59 | 46.35547 | -98.4736 | none | CS | 0 |
| 2021 | 60 | 46.35545 | -98.3613 | none | CS | 0 |
| 2021 | 61 | 46.35523 | -98.2534 | none | CS | 0 |
| 2021 | 62 | 46.35628 | -98.15 | none | CS | 0 |
| 2021 | 63 | 46.38244 | -98.0767 | none | CS | 0 |
| 2021 | 64 | 46.44208 | -98.0342 | none | CS | 0 |
| 2021 | 65 | 46.4419 | -97.9296 | none | CS | 0 |
| 2021 | 66 | 46.44193 | -97.8253 | none | CS | 0 |
| 2021 | 67 | 46.44183 | -97.7048 | none | CS | 0 |
| 2021 | 68 | 46.5001 | -97.6789 | none | CS | 0 |
| 2021 | 69 | 46.58712 | -97.679 | none | CS | 0 |
| 2021 | 70 | 46.93447 | -96.8489 | none | CS | 0 |
| 2021 | 71 | 46.9776 | -96.883 | none | CS | 0 |
| 2021 | 72 | 47.05008 | -96.9382 | none | CS | 0 |
| 2021 | 73 | 47.1516 | -96.9698 | none | CS | 0 |
| 2021 | 74 | 47.23837 | -97.0071 | none | CS | 0 |
| 2021 | 75 | 47.35287 | -97.0438 | Null | CS | 0.22 |
| 2021 | 76 | 47.41548 | -97.0624 | none | CS | 0 |
| 2021 | 77 | 47.49615 | -97.07 | none | CS | 0 |
| 2021 | 78 | 47.49817 | -97.0843 | Null | CS | 0.04 |
| 2021 | 79 | 47.49788 | -97.1992 | none | CS | 0 |
| 2021 | 80 | 47.49853 | -97.3242 | none | CS | 0 |
| 2021 | 81 | 47.50052 | -97.4048 | LL + | CS | 0.1 |
| 2021 | 82 | 47.52733 | -97.4839 | none | CS | 0 |
| 2021 | 83 | 47.52755 | -97.591 | none | CS | 0 |
| 2021 | 84 | 47.52097 | -97.7047 | none | CS | 0 |
| 2021 | 85 | 47.51223 | -97.8357 | Null | CS | 0.1 |
| 2021 | 86 | 46.86298 | -96.862 | RR + | CS | 0.15 |
| 2021 | 87 | 46.63027 | -96.8304 | LL + | CS | 0.17 |
| 2021 | 88 | 46.63025 | -96.9495 | none | CS | 0 |
| 2021 | 89 | 46.63097 | -96.9994 | LL+ ; RR+ | CS | 0.02 |
| 2021 | 90 | 46.63002 | -97.0735 | none | CS | 0 |
| 2021 | 91 | 46.62963 | -97.1784 | none | CS | 0 |
| 2021 | 92 | 46.62945 | -97.2887 | none | CS | 0 |
| 2021 | 93 | 46.62948 | -97.3673 | none | CS | 0 |
| 2021 | 94 | 46.62958 | -97.4906 | Null | CS | 0.64 |
| 2021 | 95 | 46.62968 | -97.6015 | LL + | CS | 1.75 |
| 2021 | 96 | 46.62998 | -97.7421 | LL + | CS | 0.01 |
| 2021 | 97 | 46.6301 | -97.8464 | LL + | CS | 0.19 |
| 2021 | 98 | 46.63073 | -97.9414 | LL + | CS | 0.21 |
| 2021 | 99 | 46.63097 | -98.0793 | LL + | CS | 0.2 |
| 2021 | 100 | 46.63118 | -98.1804 | LL + | CS | 0.01 |
| 2021 | 101 | 46.63127 | -98.2919 | none | CS | 0 |
| 2021 | 102 | 46.63127 | -98.3971 | none | CS | 0 |
| 2021 | 103 | 46.63107 | -98.5084 | none | CS | 0 |
| 2021 | 104 | 46.63097 | -98.6077 | none | CS | 0 |
| 2021 | 105 | 46.63097 | -98.7065 | LL + | CS | 0.03 |
| 2021 | 106 | 46.62997 | -97.6116 | LL + | CS | 10 |
| 2021 | 107 | 46.8983 | -102.796 | none | CS | 0 |
| 2021 | 108 | 46.89287 | -102.832 | none | CS | 0 |
| 2021 | 109 | 46.89157 | -102.89 | none | CS | 0 |
| 2021 | 110 | 46.88968 | -103 | none | CS | 0 |
| 2021 | 111 | 46.89847 | -103.19 | LL + | CS | 0.01 |
| 2021 | 112 | 46.81818 | -103.19 | none | CS | 0 |
| 2021 | 113 | 46.74567 | -103.19 | none | CS | 0 |
| 2021 | 114 | 46.67332 | -103.19 | none | CS | 0 |
| 2021 | 115 | 46.59837 | -103.196 | none | CS | 0 |
| 2021 | 116 | 46.5259 | -103.196 | none | CS | 0 |
| 2021 | 117 | 46.48253 | -193.238 | none | CS | 0 |
| 2021 | 118 | 46.4825 | -103.353 | none | CS | 0 |
| 2021 | 119 | 46.48248 | -103.353 | none | CS | 0 |
| 2021 | 120 | 46.52617 | -102.978 | none | CS | 0 |
| 2021 | 121 | 46.52827 | -102.868 | LL + | CS | 1.6 |
| 2021 | 122 | 46.45568 | -102.864 | Null | CS | 1.01 |
| 2021 | 123 | 46.37575 | -102.863 | LL + | CS | 0.77 |
| 2021 | 124 | 46.41233 | -102.759 | none | CS | 0 |
| 2021 | 125 | 46.41288 | -102.655 | none | CS | 0 |
| 2021 | 126 | 46.4121 | -102.55 | Null | CS | 0.02 |
| 2021 | 127 | 46.45567 | -102.864 | none | CS | 0 |
| 2021 | 128 | 46.29793 | -102.465 | none | CS | 0 |
| 2021 | 129 | 46.36372 | -102.36 | none | CS | 0 |
| 2021 | 130 | 46.42193 | -102.318 | Null | CS | 0.02 |
| 2021 | 131 | 46.50162 | -102.318 | none | CS | 0 |
| 2021 | 132 | 46.3705 | -102.206 | none | CS | 0 |
| 2021 | 133 | 46.36998 | -102.095 | none | CS | 0 |
| 2021 | 134 | 46.37057 | -101.977 | none | CS | 0 |
| 2021 | 135 | 46.34262 | -101.962 | none | CS | 0 |
| 2021 | 136 | 46.2694 | -101.957 | none | CS | 0 |
| 2021 | 137 | 46.40333 | -101.869 | none | CS | 0 |
| 2021 | 138 | 46.3426 | -101.962 | none | CS | 0 |
| 2021 | 139 | 46.36733 | -101.962 | none | CS | 0 |
| 2021 | 140 | 46.41537 | -101.896 | none | CS | 0 |
| 2021 | 141 | 46.422 | -101.463 | none | CS | 0 |
| 2021 | 142 | 46.44377 | -101.362 | none | CS | 0 |
| 2021 | 143 | 46.44835 | -101.258 | none | CS | 0 |
| 2021 | 144 | 46.45825 | -101.164 | Null | CS | 0.06 |
| 2021 | 145 | 46.46548 | -101.069 | none | CS | 0 |
| 2021 | 146 | 46.47263 | -100.965 | Null | CS | 0.01 |
| 2021 | 147 | 46.50697 | -100.903 | none | CS | 0 |
| 2021 | 148 | 46.58253 | -100.903 | none | CS | 0 |
| 2021 | 149 | 46.65633 | -100.905 | Null | CS | 0.02 |
| 2021 | 150 | 46.72937 | -100.901 | none | CS | 0 |
| 2021 | 151 | 46.80523 | -100.906 | none | CS | 0 |
| 2021 | 152 | 48.14042 | -103.724 | LL + | CS | 0.04 |
| 2021 | 153 | 48.14035 | -103.833 | none | CS | 0 |
| 2021 | 154 | 48.14679 | -103.606 | none | CS | 0 |
| 2021 | 155 | 48.15428 | -103.518 | none | CS | 0 |
| 2021 | 156 | 48.1544 | -103.41 | none | CS | 0 |
| 2021 | 157 | 48.15277 | -103.298 | none | CS | 0 |
| 2021 | 158 | 48.17627 | -103.206 | none | CS | 0 |
| 2021 | 159 | 48.17258 | -103.109 | none | CS | 0 |
| 2021 | 160 | 48.1974 | -103.035 | none | CS | 0 |
| 2021 | 161 | 48.1951 | -102.926 | none | CS | 0 |
| 2021 | 162 | 48.20453 | -102.821 | none | CS | 0 |
| 2021 | 163 | 48.21137 | -102.715 | none | CS | 0 |
| 2021 | 164 | 48.19683 | -102.613 | none | CS | 0 |
| 2021 | 165 | 48.1175 | -102.613 | none | CS | 0 |
| 2021 | 166 | 48.05038 | -102.593 | none | CS | 0 |
| 2021 | 167 | 48.02317 | -102.516 | none | CS | 0 |
| 2021 | 168 | 47.98017 | -102.483 | Null | CS | 1.38 |
| 2021 | 169 | 47.60718 | -103.258 | none | CS | 0 |
| 2021 | 170 | 47.8774 | -103.65 | none | CS | 0 |
| 2021 | 171 | 47.8771 | -103.862 | none | CS | 0 |
| 2021 | 172 | 47.80863 | -103.648 | none | CS | 0 |
| 2021 | 173 | 47.80485 | -103.537 | none | CS | 0 |
| 2021 | 174 | 47.80468 | -103.427 | none | CS | 0 |
| 2021 | 175 | 47.79945 | -103.317 | none | CS | 0 |
| 2021 | 176 | 47.7613 | -103.262 | none | CS | 0 |
| 2021 | 177 | 47.70005 | -103.283 | none | CS | 0 |
| 2021 | 178 | 47.70005 | -103.283 | none | CS | 0 |
| 2021 | 179 | 47.46363 | -103.247 | none | CS | 0 |
| 2021 | 180 | 47.39165 | -103.243 | none | CS | 0 |
| 2021 | 181 | 47.34302 | -103.163 | none | CS | 0 |
| 2021 | 182 | 47.35762 | -103.056 | none | CS | 0 |
| 2021 | 183 | 47.35752 | -102.95 | none | CS | 0 |
| 2021 | 184 | 47.3576 | -102.83 | none | CS | 0 |
| 2021 | 185 | 47.3574 | -102.727 | LL + | CS | 0.01 |
| 2021 | 186 | 47.34965 | -102.623 | none | CS | 0 |
| 2021 | 187 | 47.34242 | -102.503 | none | CS | 0 |
| 2021 | 188 | 47.3426 | -102.397 | none | CS | 0 |
| 2021 | 189 | 47.3426 | -102.315 | none | CS | 0 |
| 2021 | 190 | 47.31062 | -102.219 | none | CS | 0 |
| 2021 | 191 | 47.31375 | -102.325 | none | CS | 0 |
| 2021 | 192 | 47.30223 | -101.936 | none | CS | 0 |
| 2021 | 193 | 47.29168 | -101.86 | none | CS | 0 |
| 2021 | 194 | 47.29873 | -101.75 | none | CS | 0 |
| 2021 | 195 | 47.29928 | -101.587 | LL + | CS | 0.01 |
| 2021 | 196 | 47.29917 | -101.48 | none | CS | 0 |
| 2021 | 197 | #VALUE! | -101.374 | none | CS | 0 |
| 2021 | 198 | 47.24345 | -101.296 | none | CS | 0 |
| 2021 | 199 | 47.24532 | -101.179 | none | CS | 0 |
| 2021 | 200 | 47.26395 | -101.076 | none | CS | 0 |
| 2021 | 201 | 47.29002 | -101.02 | none | CS | 0 |
| 2021 | 202 | 47.24175 | -100.956 | none | CS | 0 |
| 2021 | 203 | 47.1894 | -100.883 | none | CS | 0 |
| 2021 | 204 | 47.15982 | -100.795 | none | CS | 0 |
| 2021 | 205 | 47.08447 | -100.792 | none | CS | 0 |
| 2021 | 206 | 46.96838 | -100.79 | none | CS | 0 |
| 2021 | 207 | 46.90078 | -100.774 | none | CS | 0 |
| 2021 | 208 | 46.89633 | -102.81 | LL + | CS | 0.1 |
| 2021 | 209 | 46.89073 | -102.89 | LL + | CS | 0.1 |
| 2021 | 210 | 46.8886 | -102.999 | none | CS | 0 |
| 2021 | 211 | 46.88773 | -103.001 | LL + | CS | 0.05 |
| 2021 | 212 | 46.89098 | -103.082 | LL + | CS | 0.19 |
| 2021 | 213 | 46.88708 | -102.736 | LL + | CS | 0.04 |
| 2021 | 214 | 46.87693 | -102.623 | none | CS | 0 |
| 2021 | 215 | 46.87647 | -102.509 | none | CS | 0 |
| 2021 | 216 | 46.87713 | -102.37 | LL + | CS | 0.11 |
| 2021 | 217 | 46.85643 | -102.245 | LL + | CS | 2.54 |
| 2021 | 218 | 46.85643 | -102.245 | RR + | CS | 0.2 |
| 2021 | 219 | 46.86287 | -101.993 | LL + | CS | 0.43 |
| 2021 | 220 | 46.86295 | -101.993 | LL + | CS | 0.12 |
| 2021 | 221 | 46.86175 | -101.841 | LL + | CS | 0.08 |
| 2021 | 222 | 46.86187 | -101.639 | none | CS | 0 |
| 2021 | 223 | 46.86128 | -101.513 | Null | CS | 0.06 |
| 2021 | 224 | 46.86108 | -101.394 | none | CS | 0 |
| 2021 | 225 | 46.86108 | -101.283 | LL + | CS | 0.36 |
| 2021 | 226 | 46.86068 | -101.157 | LL + | CS | 0.33 |
| 2021 | 227 | 46.86288 | -101.009 | LL + | CS | 0.13 |
| 2021 | 228 | 46.84123 | -100.894 | LL + | CS | 0.08 |
| 2021 | 229 | 46.83617 | -100.29 | LL + | CS | 0.18 |
| 2021 | 230 | 46.86452 | -100.141 | none | CS | 0 |
| 2021 | 231 | 46.86438 | -100.036 | LL + | CS | 0.4 |
| 2021 | 232 | 46.86418 | -99.9167 | RR + | CS | 0.62 |
| 2021 | 233 | 46.8756 | -99.8065 | none | CS | 0 |
| 2021 | 234 | 46.88062 | -99.6385 | LL + | CS | 0.18 |
| 2021 | 235 | 46.8797 | -99.4785 | none | CS | 0 |
| 2021 | 236 | 46.87735 | -99.3894 | RR + | CS | 3.15 |
| 2021 | 237 | 46.87967 | -99.4785 | none | CS | 0 |
| 2021 | 238 | 46.8868 | -99.1539 | none | CS | 0 |
| 2021 | 239 | 46.8888 | -99.0999 | LL + | CS | 6.64 |
| 2021 | 240 | 46.89168 | -98.9889 | LL + | CS | 0 |
| 2021 | 241 | 46.89225 | -98.8523 | LL + | CS | 0.38 |
| 2021 | 242 | 46.89172 | -98.9511 | LL + | CS | 0.04 |
| 2021 | 243 | 46.89172 | -98.9511 | none | CS | 0 |
| 2021 | 244 | 46.89183 | -98.0807 | LL + | CS | 0.01 |
| 2021 | 245 | 46.94625 | -98.734 | none | CS | 0 |
| 2021 | 246 | 46.00858 | -98.7782 | none | CS | 0 |
| 2021 | 247 | 47.07378 | -98.8345 | LL + | CS | 0.07 |
| 2021 | 248 | 47.14295 | -98.888 | LL + | CS | 0.04 |
| 2021 | 249 | 47.21122 | -98.9409 | LL + | CS | 0.04 |
| 2021 | 250 | 47.28382 | -98.9975 | LL + | CS | 0.04 |
| 2021 | 251 | 47.34882 | -99.0481 | none | CS | 0 |
| 2021 | 252 | 47.41593 | -99.1006 | none | CS | 0 |
| 2021 | 253 | 47.45668 | -99.2582 | LL + | CS | 0.03 |
| 2021 | 254 | 47.45767 | -99.374 | none | CS | 0 |
| 2021 | 255 | 47.45778 | -99.5011 | LL + | CS | 0 |
| 2021 | 256 | 47.4578 | -99.6293 | none | CS | 0 |
| 2021 | 257 | 47.4578 | -99.6293 | none | CS | 0 |
| 2021 | 258 | 47.44335 | -100.033 | none | CS | 0 |
| 2021 | 259 | 47.46622 | -100.118 | none | CS | 0 |
| 2021 | 260 | 47.48688 | -100.225 | none | CS | 0 |
| 2021 | 261 | 47.4872 | -100.353 | none | CS | 0 |
| 2021 | 262 | 47.48195 | -100.481 | none | CS | 0 |
| 2021 | 263 | 47.46728 | -100.609 | none | CS | 0 |
| 2021 | 264 | 47.4662 | -100.118 | none | CS | 0 |
| 2021 | 265 | 47.4662 | -100.118 | none | CS | 0 |
| 2021 | 266 | 47.46008 | -100.91 | LL + | CS | 0.01 |
| 2021 | 267 | 47.45842 | -101.035 | Null | CS | 0.02 |
| 2021 | 268 | 47.44793 | -101.122 | none | CS | 0 |
| 2021 | 269 | 47.44793 | -101.122 | none | CS | 0 |
| 2021 | 270 | 47.38595 | -101.1 | none | CS | 0 |
| 2021 | 271 | 47.30378 | -101.049 | none | CS | 0 |
| 2021 | 272 | 47.14247 | -100.791 | none | CS | 0 |
| 2021 | 273 | 47.14233 | -100.686 | LL + | CS | 0.12 |
| 2021 | 274 | 47.14227 | -100.58 | Null | CS | 0.02 |
| 2021 | 275 | 47.14223 | -100.464 | none | CS | 0 |
| 2021 | 276 | 47.14177 | -100.347 | LL + | CS | 0.39 |
| 2021 | 277 | 47.14132 | -100.229 | none | CS | 0 |
| 2021 | 278 | 47.14108 | -100.114 | none | CS | 0 |
| 2021 | 279 | 47.14082 | -99.9879 | LL + | CS | 0.03 |
| 2021 | 280 | 47.14023 | -99.8837 | none | CS | 0 |
| 2021 | 281 | 47.14027 | -99.7667 | LL + | CS | 0.02 |
| 2021 | 282 | 47.14125 | -99.6402 | none | CS | 0 |
| 2021 | 283 | 47.13957 | -99.5235 | none | CS | 0 |
| 2021 | 284 | 47.1392 | -99.4182 | none | CS | 0 |
| 2021 | 285 | 47.14373 | -99.3127 | none | CS | 0 |
| 2021 | 286 | 47.15342 | -99.2071 | Null | CS | 0.01 |
| 2021 | 287 | 47.1678 | -99.103 | none | CS | 0 |
| 2021 | 288 | 47.16782 | -98.9743 | none | CS | 0 |
| 2021 | 289 | 46.63095 | -98.7066 | none | CS | 0 |
| 2021 | 290 | 46.63083 | -98.8185 | none | CS | 0 |
| 2021 | 291 | 46.63075 | -48.9239 | none | CS | 0 |
| 2021 | 292 | 46.63028 | -99.0368 | none | CS | 0 |
| 2021 | 293 | 46.63077 | -99.1402 | none | CS | 0 |
| 2021 | 294 | 46.63117 | -99.2461 | Null | CS | 0.01 |
| 2021 | 295 | 46.6315 | -99.3453 | none | CS | 0 |
| 2021 | 296 | 46.56693 | -99.372 | none | CS | 0 |
| 2021 | 297 | 46.5147 | -99.4034 | none | CS | 0 |
| 2021 | 298 | 46.51355 | -99.5109 | none | CS | 0 |
| 2021 | 299 | 46.51475 | -99.6204 | none | CS | 0 |
| 2021 | 300 | 46.51547 | -99.727 | none | CS | 0 |
| 2021 | 301 | 46.49805 | -99.8323 | none | CS | 0 |
| 2021 | 302 | 46.50153 | -99.9376 | none | CS | 0 |
| 2021 | 303 | 46.48735 | -100.023 | none | CS | 0 |
| 2021 | 304 | 46.48753 | -100.127 | none | CS | 0 |
| 2021 | 305 | 46.48783 | -100.232 | none | CS | 0 |
| 2021 | 306 | 46.51668 | -100.294 | none | CS | 0 |
| 2021 | 307 | 46.58972 | -100.293 | LL + | CS | 0.01 |
| 2021 | 308 | 46.66293 | -100.291 | none | CS | 0 |
| 2021 | 309 | 46.73537 | -100.29 | none | CS | 0 |
| 2021 | 310 | 46.80797 | -100.289 | none | CS | 0 |
| 2021 | 311 | 47.4417 | -97.9429 | LL + | CS | 0.03 |
| 2021 | 312 | 47.44227 | -98.0496 | none | CS | 0 |
| 2021 | 313 | 47.44262 | -98.1561 | LL + | CS | 0.01 |
| 2021 | 314 | 47.44247 | -98.2652 | none | CS | 0 |
| 2021 | 315 | 47.4425 | -98.3716 | Null | CS | 0.01 |
| 2021 | 316 | 47.44727 | -98.4855 | none | CS | 0 |
| 2021 | 317 | 47.45688 | -98.516 | none | CS | 0 |
| 2021 | 318 | 47.45715 | -98.7087 | none | CS | 0 |
| 2021 | 319 | 47.45722 | -98.8196 | none | CS | 0 |
| 2021 | 320 | 47.45715 | -98.9476 | LL + | CS | 0.01 |
| 2021 | 322 | 47.45732 | -99.0639 | none | CS | 0 |
| 2021 | 323 | 47.67772 | -99.1253 | LL + | CS | 0.12 |
| 2021 | 324 | 47.68717 | -99.0399 | none | CS | 0 |
| 2021 | 325 | 47.68705 | -98.9325 | none | CS | 0 |
| 2021 | 326 | 47.68702 | -98.8087 | none | CS | 0 |
| 2021 | 327 | 47.68692 | -98.7181 | none | CS | 0 |
| 2021 | 328 | 47.68787 | -98.6113 | none | CS | 0 |
| 2021 | 329 | 47.74485 | -98.5899 | none | CS | 0 |
| 2021 | 330 | 47.80373 | -98.5655 | none | CS | 0 |
| 2021 | 331 | 47.80262 | -98.4614 | none | CS | 0 |
| 2021 | 332 | 47.80078 | -98.3545 | none | CS | 0 |
| 2021 | 333 | 47.78822 | -98.2477 | none | CS | 0 |
| 2021 | 334 | 47.75642 | -98.1611 | none | CS | 0 |
| 2021 | 335 | 47.745 | -98.0547 | none | CS | 0 |
| 2021 | 336 | 47.74467 | -97.9468 | LL + | CS | 0.01 |
| 2021 | 337 | 47.74463 | -97.8392 | none | CS | 0 |
| 2021 | 338 | 47.74463 | -97.731 | none | CS | 0 |
| 2021 | 339 | 47.74438 | -97.6249 | LL + | CS | 0.1 |
| 2021 | 340 | #VALUE! | -97.5166 | LL + | CS | 0.7 |
| 2021 | 341 | 47.74428 | -97.4096 | none | CS | 0 |
| 2021 | 342 | 47.74578 | -97.3046 | none | CS | 0 |
| 2021 | 343 | 47.7736 | -97.23 | LL + | CS | 0.01 |
| 2021 | 344 | 47.7736 | -97.0798 | Null | CS | 0.22 |
| 2021 | 345 | 48.76111 | -98.3672 | LL + | CS | 0.38 |
| 2021 | 346 | 48.76115 | -98.2556 | none | CS | 0 |
| 2021 | 347 | 48.76127 | -98.143 | LL + | CS | 0.05 |
| 2021 | 348 | 48.76098 | -98.0297 | Null | CS | 0.1 |
| 2021 | 349 | 48.76117 | -97.9278 | RR + | CS | 0.25 |
| 2021 | 350 | 48.7611 | -97.8223 | LL + | CS | 0.07 |
| 2021 | 351 | 48.77438 | -97.7125 | LL + | CS | 0.03 |
| 2021 | 352 | 48.79627 | -97.6227 | LL + | CS | 1.1 |
| 2021 | 353 | 48.80448 | -97.5236 | LL + | CS | 0.41 |
| 2021 | 354 | 48.80448 | -97.5236 | LL + | CS | 0.16 |
| 2021 | 355 | 48.80342 | -97.305 | LL + | CS | 1.07 |
| 2021 | 356 | 48.80353 | -97.2332 | LL + | CS | 7.99 |
| 2021 | 357 | 48.57128 | -97.189 | LL + | CS | 0.55 |
| 2021 | 358 | 48.57165 | -97.3214 | LL + | CS | 0.06 |
| 2021 | 359 | 48.57375 | -97.4365 | Null | CS | 0.09 |
| 2021 | 360 | 48.60103 | -97.5018 | none | CS | 0 |
| 2021 | 361 | 48.60122 | -97.6228 | LL + | CS | 0.16 |
| 2021 | 362 | 48.60118 | -97.7428 | Null | CS | 0.09 |
| 2021 | 363 | 48.6011 | -97.855 | LL + | CS | 0.05 |
| 2021 | 364 | 48.63007 | -97.9208 | Null | CS | 0.17 |
| 2021 | 365 | 48.63015 | -98.0345 | LL + | CS | 0.49 |
| 2021 | 366 | 48.63018 | -98.1554 | Null | CS | 0.01 |
| 2021 | 367 | 48.63065 | -98.2839 | LL + | CS | 0.18 |
| 2021 | 368 | 48.63068 | -98.284 | LL + | CS | 0.04 |
| 2021 | 369 | 48.7614 | -98.5845 | LL + | CS | 0.31 |
| 2021 | 370 | 48.76145 | -98.4745 | Null | CS | 0.03 |
| 2021 | 371 | 48.76153 | -98.8121 | Null | CS | 0.03 |
| 2021 | 372 | 48.78475 | -98.8909 | LL + | CS | 0.03 |
| 2021 | 373 | 48.7906 | -99.0008 | LL + | CS | 0.09 |
| 2021 | 374 | 48.79045 | -99.1233 | LL + | CS | 0.03 |
| 2021 | 375 | 48.79125 | -99.2379 | Null | CS | 0.07 |
| 2021 | 376 | 48.833 | -99.3283 | LL + | CS | 0.07 |
| 2021 | 377 | 48.83397 | -99.3293 | LL + | CS | 0.07 |
| 2021 | 378 | 48.86308 | -99.4167 | Null | CS | 0.02 |
| 2021 | 379 | 48.84951 | -99.6452 | none | CS | 0 |
| 2021 | 380 | 48.82713 | -99.7783 | LL + | CS | 0.01 |
| 2021 | 381 | 48.80575 | -99.8839 | none | CS | 0 |
| 2021 | 382 | 48.80442 | -99.9966 | none | CS | 0 |
| 2021 | 383 | 48.80613 | -100.129 | LL + | CS | 0.01 |
| 2021 | 384 | 48.8061 | -100.266 | RR + | CS | 0.05 |
| 2021 | 385 | 48.67555 | -100.297 | LL + | CS | 0.1 |
| 2021 | 386 | 48.75873 | -100.336 | none | CS | 0 |
| 2021 | 387 | 48.67577 | -100.054 | none | CS | 0 |
| 2021 | 388 | 48.6755 | -99.939 | Null | CS | 0.02 |
| 2021 | 389 | 48.66088 | -99.8313 | Null | CS | 0.09 |
| 2021 | 390 | 48.66072 | -99.7106 | Null | CS | 0.03 |
| 2021 | 391 | 48.64402 | -99.6225 | LL + | CS | 0.05 |
| 2021 | 392 | 48.63177 | -99.5171 | LL + | CS | 0.14 |
| 2021 | 393 | 48.63177 | -99.5171 | LL + | CS | 0.13 |
| 2021 | 394 | 48.63145 | -99.2822 | LL + | CS | 0.04 |
| 2021 | 395 | 48.63132 | -99.1661 | none | CS | 0 |
| 2021 | 396 | 48.63103 | -99.0357 | none | CS | 0 |
| 2021 | 397 | 48.63107 | -98.922 | none | CS | 0 |
| 2021 | 398 | 48.63067 | -98.8064 | none | CS | 0 |
| 2021 | 399 | 48.63065 | -98.6885 | LL + | CS | 0.2 |
| 2021 | 400 | 48.6302 | -98.5212 | LL + | CS | 0.02 |
| 2021 | 401 | 48.4132 | -98.3831 | none | CS | 0 |
| 2021 | 402 | 48.413 | -98.2703 | LL + | CS | 0.04 |
| 2021 | 403 | 48.41293 | -98.159 | LL + | CS | 0.01 |
| 2021 | 404 | 48.40618 | -98.0357 | none | CS | 0 |
| 2021 | 405 | 48.39825 | -97.9055 | none | CS | 0 |
| 2021 | 406 | 48.39818 | -97.7966 | Null | CS | 0.05 |
| 2021 | 407 | 48.39808 | -97.6882 | LL + | CS | 0.01 |
| 2021 | 408 | 48.41258 | -97.5847 | LL + | CS | 0.03 |
| 2021 | 409 | 48.41257 | -97.5845 | Null | CS | 0.19 |
| 2021 | 410 | 48.41257 | -97.4119 | LL + | CS | 2.33 |
| 2021 | 411 | 48.41197 | -97.2393 | LL + | CS | 0.23 |
| 2021 | 412 | 48.41198 | -97.192 | RR + | CS | 0.02 |
| 2021 | 413 | 47.93065 | -97.4905 | LL + | CS | 0.23 |
| 2021 | 414 | 47.94702 | -97.5849 | Null | CS | 0.15 |
| 2021 | 415 | 47.94753 | -97.695 | none | CS | 0 |
| 2021 | 416 | 47.98445 | -97.7913 | none | CS | 0 |
| 2021 | 417 | 48.00553 | -97.8929 | none | CS | 0 |
| 2021 | 418 | 48.00838 | -98.0057 | LL + | CS | 0.43 |
| 2021 | 419 | 48.02083 | -98.1138 | LL + | CS | 0.07 |
| 2021 | 420 | 48.02187 | -98.2224 | LL + | CS | 0.49 |
| 2021 | 421 | 48.02203 | -98.2219 | LL + | CS | 0.15 |
| 2021 | 422 | 48.0356 | -98.4442 | none | CS | 0 |
| 2021 | 423 | 48.05032 | -98.552 | none | CS | 0 |
| 2021 | 424 | 48.0502 | -98.67 | Null | CS | 0.03 |
| 2021 | 425 | 48.06125 | -98.7786 | none | CS | 0 |
| 2021 | 426 | 48.09838 | -98.8351 | LL + | CS | 1 |
| 2021 | 427 | 48.13468 | -98.9091 | LL + | CS | 3.54 |
| 2021 | 428 | 48.17678 | -99.0057 | LL + | CS | 0.24 |
| 2021 | 429 | 48.22467 | -99.1031 | Null | CS | 0.09 |
| 2021 | 430 | 48.26333 | -99.1953 | Null | CS | 0.01 |
| 2021 | 431 | 48.2789 | -99.3086 | none | CS | 0 |
| 2021 | 432 | 48.28752 | -99.4192 | LL + | CS | 0.04 |
| 2021 | 433 | 48.30693 | -99.5341 | none | CS | 0 |
| 2021 | 434 | 48.33108 | -99.6415 | LL + | CS | 0.02 |
| 2021 | 435 | 48.34032 | -99.751 | LL + | CS | 0.04 |
| 2021 | 436 | 48.35458 | -99.8581 | LL + | CS | 0.05 |
| 2021 | 437 | 48.35497 | -99.9771 | none | CS | 0 |
| 2021 | 438 | 48.52373 | -100.198 | LL + | CS | 0.01 |
| 2021 | 439 | 48.50153 | -100.105 | none | CS | 0 |
| 2021 | 440 | 48.50152 | -99.9937 | LL + | CS | 0.01 |
| 2021 | 441 | 48.50143 | -99.8849 | none | CS | 0 |
| 2021 | 442 | 48.50145 | -99.7724 | none | CS | 0 |
| 2021 | 443 | 48.50147 | -99.6602 | none | CS | 0 |
| 2021 | 444 | 48.50137 | -99.5448 | none | CS | 0 |
| 2021 | 445 | 48.48675 | -99.4282 | none | CS | 0 |
| 2021 | 446 | 48.48672 | -99.3031 | none | CS | 0 |
| 2021 | 447 | 48.48658 | -99.1824 | none | CS | 0 |
| 2021 | 448 | 48.4865 | -99.0689 | none | CS | 0 |
| 2021 | 449 | 48.48627 | -98.9552 | LL + | CS | 0.01 |
| 2021 | 450 | 48.48627 | -98.9552 | none | CS | 0 |
| 2021 | 451 | 48.41365 | -98.8357 | LL + | CS | 0.02 |
| 2021 | 452 | 48.41343 | -98.721 | LL + | CS | 0.04 |
| 2021 | 453 | 48.41345 | -98.6049 | none | CS | 0 |
| 2021 | 454 | 48.41332 | -98.4923 | LL + | CS | 0.03 |
| 2021 | 455 | 47.64628 | -101.844 | none | CS | 0 |
| 2021 | 456 | 47.64643 | -101.737 | RR + | CS | 0.01 |
| 2021 | 457 | 48.6883 | -102.082 | Null | CS | 0.01 |
| 2021 | 458 | 47.64255 | -101.515 | none | CS | 0 |
| 2021 | 459 | 47.64255 | -101.404 | Null | CS | 0.01 |
| 2021 | 460 | 47.64698 | -101.292 | none | CS | 0 |
| 2021 | 461 | 47.71787 | -101.293 | RR + | CS | 0.01 |
| 2021 | 462 | 47.81885 | -101.29 | Null | CS | 0.12 |
| 2021 | 463 | 47.81913 | -101.185 | LL + | CS | 1.85 |
| 2021 | 464 | 47.8191 | -101.075 | none | CS | 0 |
| 2021 | 465 | 47.81915 | -100.962 | LL + | CS | 0.01 |
| 2021 | 466 | 47.83372 | -100.864 | none | CS | 0 |
| 2021 | 467 | 47.82675 | -100.757 | none | CS | 0 |
| 2021 | 468 | 47.83327 | -100.65 | none | CS | 0 |
| 2021 | 469 | 47.83313 | -100.548 | none | CS | 0 |
| 2021 | 470 | 47.89113 | -100.521 | none | CS | 0 |
| 2021 | 471 | 48.2288 | -101.204 | LL + | CS | 0.14 |
| 2021 | 472 | 48.23975 | -101.097 | LL + | CS | 0.01 |
| 2021 | 473 | 48.23967 | -100.973 | none | CS | 0 |
| 2021 | 474 | 48.24015 | -100.849 | LL + | CS | 0.01 |
| 2021 | 475 | 48.2816 | -100.767 | Null | CS | 0.02 |
| 2021 | 476 | 48.28162 | -100.767 | none | CS | 0 |
| 2021 | 477 | 48.31163 | -100.54 | none | CS | 0 |
| 2021 | 478 | 48.31165 | -100.432 | LL + | CS | 0.04 |
| 2021 | 479 | 48.35477 | -100.346 | LL + | CS | 0.05 |
| 2021 | 480 | 48.35528 | -100.237 | LL + | CS | 0.06 |
| 2021 | 481 | 48.35485 | -100.126 | LL + | CS | 0.14 |
| 2021 | 482 | 48.35508 | -0.9947 | none | CS | 0 |
| 2021 | 483 | 48.61745 | -100.298 | Null | CS | 0.44 |
| 2021 | 484 | 48.82095 | -100.444 | Null | CS | 0.13 |
| 2021 | 485 | 48.82093 | -100.556 | Null | CS | 0.03 |
| 2021 | 486 | 48.82108 | -100.674 | none | CS | 0 |
| 2021 | 487 | 48.82098 | -100.786 | Null | CS | 0.01 |
| 2021 | 488 | 48.82078 | -100.908 | Null | CS | 0.07 |
| 2021 | 489 | 48.82135 | -101.017 | none | CS | 0 |
| 2021 | 490 | 48.81935 | -101.146 | none | CS | 0 |
| 2021 | 491 | 48.76322 | -101.301 | LL + | CS | 0.08 |
| 2021 | 492 | 48.76328 | -101.409 | LL + | CS | 0.02 |
| 2021 | 493 | 48.81937 | -101.146 | none | CS | 0 |
| 2021 | 494 | 48.76353 | -101.628 | none | CS | 0 |
| 2021 | 495 | 48.7635 | -101.746 | RR + | CS | 0.02 |
| 2021 | 496 | 48.76363 | -101.862 | LL + | CS | 0.02 |
| 2021 | 497 | 48.76375 | -101.978 | Null | CS | 0.08 |
| 2021 | 498 | 48.76382 | -102.087 | LL + | CS | 0.02 |
| 2021 | 499 | 48.68757 | -102.099 | LL + | CS | 0.21 |
| 2021 | 500 | 48.62788 | -102.011 | RR + | CS | 0.01 |
| 2021 | 501 | 48.55789 | -101.966 | Null | CS | 0.07 |
| 2021 | 502 | 48.54638 | -102.071 | RR + | CS | 0.4 |
| 2021 | 503 | 48.5464 | -102.182 | RR + | CS | 0.03 |
| 2021 | 504 | 48.54635 | -102.31 | RR + | CS | 0.02 |
| 2021 | 505 | 48.54635 | -102.409 | LL + | CS | 0.14 |
| 2021 | 506 | 48.56093 | -102.504 | LL + | CS | 0.04 |
| 2021 | 507 | 48.56097 | -102.612 | none | CS | 0 |
| 2021 | 508 | 48.57537 | -102.718 | none | CS | 0 |
| 2021 | 509 | 48.57531 | -102.835 | LL + | CS | 0.04 |
| 2021 | 510 | 48.57585 | -102.777 | LL + | CS | 0.17 |
| 2021 | 511 | 48.59272 | -102.908 | none | CS | 0 |
| 2021 | 512 | 48.59008 | -103.044 | none | CS | 0 |
| 2021 | 513 | 48.57563 | -103.302 | LL + | CS | 0.38 |
| 2021 | 514 | 48.5805 | -103.385 | none | CS | 0 |
| 2021 | 515 | 48.57578 | -103.484 | LL + | CS | 0.01 |
| 2021 | 516 | 48.57085 | -103.602 | LL + | CS | 0.05 |
| 2021 | 517 | 48.57875 | -103.727 | none | CS | 0 |
| 2021 | 518 | 48.89485 | -103.947 | RR + | CS | 0.01 |
| 2021 | 519 | 48.90912 | -103.827 | RR + | CS | 0.01 |
| 2021 | 520 | 48.90913 | -103.7 | LL + | CS | 0.02 |
| 2021 | 521 | 48.90877 | -103.658 | LL + | CS | 0.25 |
| 2021 | 522 | 48.90868 | -103.455 | LL + | CS | 0.01 |
| 2021 | 523 | 48.90868 | -103.333 | LL + | CS | 0.12 |
| 2021 | 524 | 48.89432 | -103.23 | Null | CS | 0.07 |
| 2021 | 525 | 48.89438 | -103.114 | LL + | CS | 0.03 |
| 2021 | 526 | 48.89438 | -103 | LL + | CS | 0.02 |
| 2021 | 527 | 48.89443 | -102.874 | LL + | CS | 0.01 |
| 2021 | 528 | 48.89447 | -102.765 | none | CS | 0 |
| 2021 | 529 | 48.8951 | -102.546 | LL + | CS | 0.07 |
| 2021 | 530 | 48.89408 | -102.656 | none | CS | 0 |
| 2021 | 531 | 48.89427 | -102.433 | LL + | CS | 0.66 |
| 2021 | 532 | 48.89425 | -102.303 | LL+ | CS | 10.04 |
| 2021 | 533 | 48.87225 | -102.24 | LL + | CS | 0.01 |
| 2021 | 534 | 48.8073 | -102.224 | LL + | CS | 0.5 |
| 2021 | 535 | 48.80717 | -102.119 | Null | CS | 0.02 |
| 2021 | 536 | 48.57247 | -102 | none | CS | 0 |
| 2021 | 537 | 48.50887 | -101.887 | none | CS | 0 |
| 2021 | 538 | 48.47083 | -101.786 | none | CS | 0 |
| 2021 | 539 | 48.43492 | -101.699 | none | CS | 0 |
| 2021 | 540 | 48.38652 | -101.607 | LL + | CS | 0.13 |
| 2021 | 541 | 48.33457 | -101.522 | Null | CS | 0.02 |
| 2021 | 542 | 48.28372 | -101.461 | Null | CS | 0.76 |
| 2021 | 543 | 48.23705 | -101.384 | LL + | CS | 0.32 |
| 2021 | 544 | 48.19462 | -101.296 | Null | CS | 0.04 |
| 2021 | 545 | 48.12442 | -101.296 | none | CS | 0 |
| 2021 | 546 | 48.04472 | -101.296 | none | CS | 0 |
| 2021 | 547 | 47.97118 | -101.294 | none | CS | 0 |
| 2021 | 548 | 47.89192 | -101.293 | none | CS | 0 |
| 2021 | 549 | 47.57442 | -101.26 | LL + | CS | 0.01 |
| 2021 | 550 | 47.51746 | -101.187 | LL + | CS | 0.01 |
| 2021 | 551 | 47.4585 | -101.126 | none | CS | 0 |
| 2021 | 552 | 47.99323 | -100.93 | LL+ ; RR+ | CS | 1.03 |
| 2021 | 553 | 47.99337 | -101.04 | RR + | CS | 0.01 |
| 2021 | 554 | 47.97887 | -101.143 | Null | CS | 0.22 |
| 2021 | 555 | 47.97885 | -101.259 | LL + | CS | 0.05 |
| 2021 | 556 | 47.97882 | -101.366 | none | CS | 0 |
| 2021 | 557 | 47.97858 | -101.48 | none | CS | 0 |
| 2021 | 558 | 47.97828 | -101.589 | none | CS | 0 |
| 2021 | 559 | 47.97822 | -101.701 | none | CS | 0 |
| 2021 | 560 | 47.96795 | -101.807 | none | CS | 0 |
| 2021 | 561 | 47.9781 | -101.923 | none | CS | 0 |
| 2021 | 562 | 47.97792 | -102.043 | none | CS | 0 |
| 2021 | 563 | 47.97793 | -102.159 | LL + | CS | 0.01 |
| 2021 | 564 | 47.97785 | -102.259 | LL + | CS | 0.6 |
| 2021 | 565 | 47.97797 | -102.386 | none | CS | 0 |
| 2021 | 566 | 47.8805 | -104.017 | none | CS | 0 |
| 2021 | 567 | 48.23062 | -103.625 | none | CS | 0 |
| 2021 | 568 | 48.30588 | -103.626 | none | CS | 0 |
| 2021 | 569 | 48.3421 | -103.561 | LL + | CS | 0.21 |
| 2021 | 570 | 48.34222 | -103.443 | LL + | CS | 0.03 |
| 2021 | 571 | 48.3422 | -103.325 | RR + | CS | 0.51 |
| 2021 | 572 | 48.34202 | -103.216 | LL + | CS | 1.59 |
| 2021 | 573 | 48.34225 | -103.102 | none | CS | 0 |
| 2021 | 574 | 48.34163 | -102.995 | LL + | CS | 0.4 |
| 2021 | 575 | 48.34177 | -102.884 | none | CS | 0 |
| 2021 | 576 | 48.32568 | -102.78 | Null | CS | 0.06 |
| 2021 | 577 | 48.31273 | -102.656 | Null | CS | 0.13 |
| 2021 | 578 | 48.31165 | -102.548 | RR + | CS | 0.18 |
| 2021 | 579 | 48.31027 | -102.435 | LL + | CS | 0.07 |
| 2021 | 580 | 48.32195 | -102.314 | LL + | CS | 0.28 |
| 2021 | 581 | 48.32698 | -102.203 | LL + | CS | 0.5 |
| 2021 | 582 | 48.3269 | -102.091 | LL + | CS | 0.28 |
| 2021 | 583 | 48.32693 | -101.979 | Null | CS | 0.99 |
| 2021 | 584 | 48.32695 | -101.867 | LL + | CS | 0.04 |
| 2021 | 585 | 48.32572 | -101.739 | LL + | CS | 0.22 |
| 2021 | 586 | 48.31255 | -101.621 | LL + | CS | 0.01 |
| 2021 | 587 | 48.0225 | -100.929 | LL+ ; RR+ | CS | 0.79 |
| 2021 | 588 | 48.02342 | -100.876 | LL + | CS | 0.52 |
| 2021 | 589 | 48.05608 | -100.938 | RR + | CS | 1.49 |
| 2021 | 590 | 48.0092 | -100.763 | LL + | CS | 2.3 |
| 2021 | 591 | 47.9861 | -100.65 | none | CS | 0 |
| 2021 | 592 | 47.95147 | -100.542 | LL + | CS | 0.02 |
| 2021 | 593 | 47.926 | -100.447 | LL + | CS | 0.07 |
| 2021 | 594 | 47.91355 | -100.333 | none | CS | 0 |
| 2021 | 595 | 47.87705 | -100.241 | none | CS | 0 |
| 2021 | 596 | 47.83653 | -100.142 | LL + | CS | 0.03 |
| 2021 | 597 | 47.80562 | -100.037 | none | CS | 0 |
| 2021 | 598 | 47.7763 | -99.9484 | LL + | CS | 0.01 |
| 2021 | 599 | 47.74005 | -99.8654 | none | CS | 0 |
| 2021 | 600 | 47.7024 | -99.7764 | none | CS | 0 |
| 2021 | 601 | 47.6685 | -99.6759 | none | CS | 0 |
| 2021 | 602 | 47.64587 | -99.5862 | none | CS | 0 |
| 2021 | 603 | 47.6461 | -99.4743 | none | CS | 0 |
| 2021 | 604 | 47.64478 | -99.3622 | none | CS | 0 |
| 2021 | 605 | 47.6456 | -99.2453 | none | CS | 0 |
| 2021 | 606 | 47.64575 | -99.1169 | none | CS | 0 |
| 2021 | 607 | 46.88435 | -98.7158 | Null | CS | 3 |
| 2021 | 608 | 47.42482 | -97.0715 | Null | CS | 0.07 |
| 2021 | 609 | 47.3527 | -97.0502 | LL + | CS | 0.02 |
| 2021 | 610 | 47.24802 | -97.0155 | Null | CS | 0.01 |
| 2021 | 611 | 47.15198 | -96.975 | none | CS | 0 |
| 2021 | 612 | 47.05058 | -96.9407 | none | CS | 0 |
| 2021 | 613 | 46.97768 | -96.8854 | Null | CS | 0.09 |
| 2021 | 614 | 46.91253 | -98.0794 | LL + | CS | 1.1 |
| 2021 | 615 | 46.91157 | -98.0229 | Null | CS | 0.21 |
| 2021 | 616 | 46.92012 | -97.9136 | none | CS | 0 |
| 2021 | 617 | 46.91977 | -97.7884 | LL + | CS | 0.27 |
| 2021 | 618 | 46.91887 | -97.6841 | LL + | CS | 0.05 |
| 2021 | 619 | 46.87563 | -97.5576 | LL + | CS | 0.4 |
| 2021 | 620 | 46.87555 | -97.4302 | Null | CS | 0.02 |
| 2021 | 621 | 46.87572 | -97.2648 | Null | CS | 0.15 |
| 2021 | 622 | 46.8765 | -97.1599 | LL + | CS | 0.03 |
| 2021 | 623 | 46.87708 | -97.0527 | none | CS | 0 |
| 2021 | 624 | 46.87632 | -96.9479 | none | CS | 0 |
| 2021 | 625 | 46.63008 | -97.9263 | LL+ | CS | 0.5 |
| 2010 | 1 | 46.09942 | -96.8359 | LL+ | JPL | 0.02 |
| 2010 | 2 | 46.05145 | -96.7284 | none | JPL | 0 |
| 2010 | 3 | 46.05097 | -96.6216 | none | JPL | 0 |
| 2010 | 4 | 46.05167 | -96.8432 | none | JPL | 0 |
| 2010 | 5 | 46.07473 | -96.9361 | none | JPL | 0 |
| 2010 | 6 | 46.06678 | -97.0353 | none | JPL | 0 |
| 2010 | 7 | 46.06783 | -97.1412 | none | JPL | 0 |
| 2010 | 8 | 46.07885 | -97.2326 | none | JPL | 0 |
| 2010 | 9 | 46.07963 | -97.3356 | none | JPL | 0 |
| 2010 | 10 | 46.07985 | -97.4394 | none | JPL | 0 |
| 2010 | 11 | 46.08035 | -97.5399 | none | JPL | 0 |
| 2010 | 12 | 46.08808 | -97.6335 | RR + | JPL | 0.05 |
| 2010 | 13 | 46.10265 | -97.7334 | none | JPL | 0 |
| 2010 | 14 | 46.10263 | -97.8366 | none | JPL | 0 |
| 2010 | 15 | 46.10983 | -97.9378 | none | JPL | 0 |
| 2010 | 16 | 46.1098 | -98.0428 | none | JPL | 0 |
| 2010 | 17 | 46.06448 | -98.0911 | none | JPL | 0 |
| 2010 | 18 | 46.00852 | -98.1167 | none | JPL | 0 |
| 2010 | 19 | 46.00858 | -98.221 | none | JPL | 0 |
| 2010 | 20 | 46.0086 | -98.3248 | none | JPL | 0 |
| 2010 | 21 | 46.009 | -98.4284 | none | JPL | 0 |
| 2010 | 22 | 46.00277 | -98.5406 | none | JPL | 0 |
| 2010 | 23 | 46.00363 | -98.6471 | none | JPL | 0 |
| 2010 | 24 | 46.02577 | -98.7227 | none | JPL | 0 |
| 2010 | 25 | 46.02563 | -98.8253 | none | JPL | 0 |
| 2010 | 26 | 46.02683 | -98.9285 | none | JPL | 0 |
| 2010 | 28 | 46.02678 | -99.0293 | none | JPL | 0 |
| 2010 | 29 | 46.02702 | -99.1309 | none | JPL | 0 |
| 2010 | 30 | 46.027 | -99.2335 | none | JPL | 0 |
| 2010 | 31 | 46.03407 | -99.337 | none | JPL | 0 |
| 2010 | 32 | 46.0346 | -99.4337 | none | JPL | 0 |
| 2010 | 33 | 46.02762 | -99.5347 | none | JPL | 0 |
| 2010 | 34 | 46.02802 | -99.6373 | none | JPL | 0 |
| 2010 | 35 | 46.0278 | -99.7423 | none | JPL | 0 |
| 2010 | 36 | 46.0136 | -99.8283 | none | JPL | 0 |
| 2010 | 37 | 46.02867 | -99.915 | none | JPL | 0 |
| 2010 | 38 | 46.02792 | -100.019 | none | JPL | 0 |
| 2010 | 39 | 46.09205 | -100.104 | none | JPL | 0 |
| 2010 | 40 | 46.14328 | -100.171 | none | JPL | 0 |
| 2010 | 41 | 46.20767 | -100.211 | none | JPL | 0 |
| 2010 | 42 | 46.25508 | -100.215 | none | JPL | 0 |
| 2010 | 43 | 46.24663 | -100.111 | none | JPL | 0 |
| 2010 | 44 | 46.24627 | -100.008 | none | JPL | 0 |
| 2010 | 45 | 46.26008 | -99.9082 | none | JPL | 0 |
| 2010 | 46 | 46.25935 | -99.8068 | none | JPL | 0 |
| 2010 | 47 | 46.25963 | -99.7068 | none | JPL | 0 |
| 2010 | 48 | 46.25928 | -99.6029 | none | JPL | 0 |
| 2010 | 49 | 46.25892 | -99.4987 | none | JPL | 0 |
| 2010 | 50 | 46.26723 | -99.3998 | none | JPL | 0 |
| 2010 | 51 | 46.28333 | -99.3252 | Null | JPL | 0.02 |
| 2010 | 52 | 46.28312 | -99.2248 | none | JPL | 0 |
| 2010 | 53 | 46.30125 | -99.1317 | none | JPL | 0 |
| 2010 | 54 | 46.312 | -99.0305 | none | JPL | 0 |
| 2010 | 55 | 46.3313 | -98.954 | none | JPL | 0 |
| 2010 | 56 | 46.35533 | -98.8817 | none | JPL | 0 |
| 2010 | 57 | 46.35525 | -98.7785 | none | JPL | 0 |
| 2010 | 58 | 46.3553 | -98.6736 | none | JPL | 0 |
| 2010 | 59 | 46.3553 | -98.5699 | none | JPL | 0 |
| 2010 | 60 | 46.3555 | -98.4655 | none | JPL | 0 |
| 2010 | 61 | 46.35547 | -98.3592 | none | JPL | 0 |
| 2010 | 62 | 46.35525 | -98.2521 | none | JPL | 0 |
| 2010 | 63 | 46.35578 | -98.1523 | none | JPL | 0 |
| 2010 | 64 | 46.38768 | -98.0764 | none | JPL | 0 |
| 2010 | 65 | 46.44245 | -98.054 | none | JPL | 0 |
| 2010 | 66 | 46.44208 | -97.9595 | none | JPL | 0 |
| 2010 | 67 | 46.4419 | -97.8551 | none | JPL | 0 |
| 2010 | 68 | 46.44208 | -97.7497 | none | JPL | 0 |
| 2010 | 69 | 46.44202 | -97.6461 | none | JPL | 0 |
| 2010 | 70 | 46.44225 | -97.541 | none | JPL | 0 |
| 2010 | 71 | 46.4793 | -97.4897 | none | JPL | 0 |
| 2010 | 72 | 46.5528 | -97.4898 | none | JPL | 0 |
| 2010 | 73 | 46.62473 | -97.4904 | RR + | JPL | 1.07 |
| 2010 | 74 | 46.62978 | -97.5898 | RR + | JPL | 0.02 |
| 2010 | 75 | 46.62743 | -97.6036 | RR + | JPL | 1 |
| 2010 | 76 | 46.63002 | -97.6875 | LL+ | JPL | 2.2 |
| 2010 | 77 | 46.63025 | -97.7898 | RR + | JPL | 2.36 |
| 2010 | 78 | 46.87758 | -97.0212 | none | JPL | 0 |
| 2010 | 79 | 46.8769 | -97.1052 | RR + | JPL | 0.03 |
| 2010 | 80 | 46.87413 | -97.2227 | RR + | JPL | 0.02 |
| 2010 | 81 | 46.87583 | -97.3494 | none | JPL | 0 |
| 2010 | 82 | 46.87587 | -97.446 | Null | JPL | 0.02 |
| 2010 | 83 | 46.876 | -97.5732 | RR + | JPL | 0.03 |
| 2010 | 84 | 46.90798 | -97.6659 | RR + | JPL | 0.02 |
| 2010 | 85 | 46.9202 | -97.8052 | RR + | JPL | 0.03 |
| 2010 | 86 | 46.92062 | -97.9262 | LL+ | JPL | 0.01 |
| 2010 | 87 | 46.91207 | -98.0336 | LL+ | JPL | 0.27 |
| 2010 | 88 | 46.87208 | -98.0809 | none | JPL | 0 |
| 2010 | 89 | 46.79923 | -98.0811 | none | JPL | 0 |
| 2010 | 90 | 46.7268 | -98.0816 | none | JPL | 0 |
| 2010 | 91 | 46.65662 | -98.0817 | LL+ | JPL | 0.15 |
| 2010 | 92 | 46.63065 | -97.9717 | LL+ ; RR+ | JPL | 0.09 |
| 2010 | 93 | 46.63025 | -97.8712 | none | JPL | 0 |
| 2010 | 94 | 46.63102 | -98.1866 | LL+ | JPL | 0.01 |
| 2010 | 95 | 46.63105 | -98.2909 | RR + | JPL | 0.01 |
| 2010 | 96 | 46.6311 | -98.3969 | none | JPL | 0 |
| 2010 | 97 | 46.6311 | -98.5023 | none | JPL | 0 |
| 2010 | 98 | 46.63098 | -98.6078 | RR + | JPL | 0.04 |
| 2010 | 99 | 46.63095 | -98.7083 | LL+ | JPL | 0.09 |
| 2010 | 100 | 46.63068 | -98.8191 | Null | JPL | 0.01 |
| 2010 | 101 | 46.63055 | -98.9228 | none | JPL | 0 |
| 2010 | 102 | 46.63063 | -99.0374 | none | JPL | 0 |
| 2010 | 103 | 46.63075 | -99.1315 | Null | JPL | 0.02 |
| 2010 | 104 | 46.56735 | -99.1417 | none | JPL | 0 |
| 2010 | 105 | 46.5294 | -99.1915 | none | JPL | 0 |
| 2010 | 106 | 46.52925 | -99.2973 | none | JPL | 0 |
| 2010 | 107 | 46.51468 | -99.3826 | none | JPL | 0 |
| 2010 | 108 | 46.51495 | -99.488 | none | JPL | 0 |
| 2010 | 109 | 46.51488 | -99.5946 | none | JPL | 0 |
| 2010 | 110 | 46.51528 | -99.6933 | none | JPL | 0 |
| 2010 | 111 | 46.51258 | -99.7986 | none | JPL | 0 |
| 2010 | 112 | 46.5014 | -99.895 | RR + | JPL | 0.05 |
| 2010 | 113 | 46.48702 | -99.9871 | none | JPL | 0 |
| 2010 | 114 | 46.48728 | -100.09 | none | JPL | 0 |
| 2010 | 115 | 46.48758 | -100.196 | none | JPL | 0 |
| 2010 | 116 | 46.49025 | -100.293 | none | JPL | 0 |
| 2010 | 117 | 46.57377 | -100.294 | none | JPL | 0 |
| 2010 | 118 | 46.64742 | -100.291 | none | JPL | 0 |
| 2010 | 119 | 46.69878 | -100.291 | LL+ | JPL | 0.11 |
| 2010 | 120 | 46.777 | -100.289 | none | JPL | 0 |
| 2010 | 121 | 46.86458 | -100.181 | none | JPL | 0 |
| 2010 | 122 | 46.86445 | -100.07 | none | JPL | 0 |
| 2010 | 123 | 46.8645 | -99.9575 | none | JPL | 0 |
| 2010 | 124 | 46.87188 | -99.855 | none | JPL | 0 |
| 2010 | 125 | 46.87835 | -99.7473 | none | JPL | 0 |
| 2010 | 126 | 46.87977 | -99.6379 | none | JPL | 0 |
| 2010 | 127 | 46.87992 | -99.492 | none | JPL | 0 |
| 2010 | 128 | 46.87792 | -99.3826 | none | JPL | 0 |
| 2010 | 129 | 46.87863 | -99.2721 | none | JPL | 0 |
| 2010 | 130 | 46.88662 | -99.1611 | none | JPL | 0 |
| 2010 | 131 | 46.8911 | -99.0693 | none | JPL | 0 |
| 2010 | 132 | 46.89212 | -98.9435 | none | JPL | 0 |
| 2010 | 133 | 46.89227 | -98.8111 | none | JPL | 0 |
| 2010 | 134 | 46.89213 | -98.679 | LL+ | JPL | 0.01 |
| 2010 | 135 | 46.97705 | -98.7498 | none | JPL | 0 |
| 2010 | 136 | 47.04135 | -98.8073 | RR + | JPL | 0.07 |
| 2010 | 137 | 47.10205 | -98.8564 | Null | JPL | 0.02 |
| 2010 | 138 | 47.16607 | -98.9059 | LL+ | JPL | 0.1 |
| 2010 | 139 | 47.16797 | -99.0127 | none | JPL | 0 |
| 2010 | 140 | 47.16567 | -99.1174 | none | JPL | 0 |
| 2010 | 141 | 47.15353 | -99.2138 | none | JPL | 0 |
| 2010 | 142 | 47.1538 | -99.3013 | none | JPL | 0 |
| 2010 | 143 | 47.1394 | -99.4105 | none | JPL | 0 |
| 2010 | 144 | 47.13983 | -99.5138 | none | JPL | 0 |
| 2010 | 145 | 47.14012 | -99.7231 | none | JPL | 0 |
| 2010 | 146 | 47.14012 | -99.7231 | none | JPL | 0 |
| 2010 | 147 | 47.1417 | -99.8297 | none | JPL | 0 |
| 2010 | 148 | 47.14007 | -99.9342 | none | JPL | 0 |
| 2010 | 149 | 47.14023 | -100.037 | none | JPL | 0 |
| 2010 | 150 | 47.14122 | -100.144 | none | JPL | 0 |
| 2010 | 151 | 47.14133 | -100.247 | none | JPL | 0 |
| 2010 | 152 | 47.14197 | -100.354 | none | JPL | 0 |
| 2010 | 153 | 47.14223 | -100.462 | none | JPL | 0 |
| 2010 | 154 | 47.14228 | -100.566 | none | JPL | 0 |
| 2010 | 155 | 47.14265 | -100.672 | none | JPL | 0 |
| 2010 | 156 | 47.14262 | -100.778 | none | JPL | 0 |
| 2010 | 157 | 47.06672 | -100.792 | none | JPL | 0 |
| 2010 | 158 | 46.99483 | -100.792 | none | JPL | 0 |
| 2010 | 159 | 46.92363 | -100.772 | none | JPL | 0 |
| 2010 | 160 | 46.82698 | -100.885 | LL+ | JPL | 0.01 |
| 2010 | 161 | 46.75242 | -100.902 | RR + | JPL | 0.01 |
| 2010 | 162 | 46.6796 | -100.903 | none | JPL | 0 |
| 2010 | 163 | 46.60833 | -100.903 | none | JPL | 0 |
| 2010 | 164 | 46.53525 | -100.903 | none | JPL | 0 |
| 2010 | 165 | 46.47188 | -100.921 | none | JPL | 0 |
| 2010 | 166 | 46.47287 | -101.027 | none | JPL | 0 |
| 2010 | 167 | 46.45475 | -101.12 | none | JPL | 0 |
| 2010 | 168 | 46.45843 | -101.238 | none | JPL | 0 |
| 2010 | 169 | 46.4439 | -101.333 | none | JPL | 0 |
| 2010 | 170 | 46.43193 | -101.432 | none | JPL | 0 |
| 2010 | 171 | 46.41502 | -101.532 | none | JPL | 0 |
| 2010 | 172 | 46.40065 | -101.618 | none | JPL | 0 |
| 2010 | 173 | 46.40093 | -101.73 | none | JPL | 0 |
| 2010 | 174 | 46.40135 | -101.828 | none | JPL | 0 |
| 2010 | 175 | 46.38317 | -101.924 | Null | JPL | 0.01 |
| 2010 | 176 | 46.33187 | -101.965 | LL+ | JPL | 0.01 |
| 2010 | 177 | 46.26087 | -101.957 | RR + | JPL | 0.06 |
| 2010 | 178 | 46.37163 | -102.068 | RR + | JPL | 0.01 |
| 2010 | 179 | 46.3703 | -102.173 | none | JPL | 0 |
| 2010 | 180 | 46.3715 | -102.278 | none | JPL | 0 |
| 2010 | 181 | 46.37813 | -102.318 | RR + | JPL | 0.25 |
| 2010 | 182 | 46.4526 | -102.318 | none | JPL | 0 |
| 2010 | 183 | 46.52347 | -102.318 | none | JPL | 0 |
| 2010 | 184 | 46.36368 | -102.398 | none | JPL | 0 |
| 2010 | 185 | 46.34633 | -102.465 | none | JPL | 0 |
| 2010 | 186 | 46.27555 | -102.477 | none | JPL | 0 |
| 2010 | 187 | 46.39183 | -102.507 | none | JPL | 0 |
| 2010 | 188 | 46.41318 | -102.6 | Null | JPL | 0.03 |
| 2010 | 189 | 46.41288 | -102.697 | none | JPL | 0 |
| 2010 | 190 | 46.41215 | -102.812 | none | JPL | 0 |
| 2010 | 191 | 46.41215 | -102.863 | RR + | JPL | 0.04 |
| 2010 | 192 | 46.33943 | -102.863 | none | JPL | 0 |
| 2010 | 193 | 46.26555 | -102.867 | none | JPL | 0 |
| 2010 | 194 | 46.48217 | -102.864 | none | JPL | 0 |
| 2010 | 195 | 46.52872 | -102.867 | RR + | JPL | 0.01 |
| 2010 | 196 | 46.52622 | -102.977 | RR + | JPL | 0.01 |
| 2010 | 197 | 46.52623 | -103.081 | RR + | JPL | 0.01 |
| 2010 | 198 | 46.52603 | -103.195 | none | JPL | 0 |
| 2010 | 199 | 46.48255 | -103.254 | none | JPL | 0 |
| 2010 | 200 | 46.48255 | -103.327 | none | JPL | 0 |
| 2010 | 201 | 46.6014 | -103.196 | none | JPL | 0 |
| 2010 | 202 | 46.67487 | -103.19 | none | JPL | 0 |
| 2010 | 203 | 46.7469 | -103.189 | none | JPL | 0 |
| 2010 | 204 | 46.81917 | -103.19 | none | JPL | 0 |
| 2010 | 205 | 46.88903 | -103.19 | LL+ | JPL | 0.01 |
| 2010 | 206 | 46.89108 | -103.093 | none | JPL | 0 |
| 2010 | 207 | 46.88708 | -102.999 | none | JPL | 0 |
| 2010 | 208 | 46.89045 | -102.886 | none | JPL | 0 |
| 2010 | 209 | 46.89588 | -102.782 | none | JPL | 0 |
| 2010 | 210 | 46.88323 | -102.706 | RR + | JPL | 0.1 |
| 2010 | 211 | 46.88132 | -102.585 | RR + | JPL | 0.22 |
| 2010 | 212 | 46.87675 | -102.473 | RR + | JPL | 1 |
| 2010 | 213 | 46.87705 | -102.394 | RR + | JPL | 0.25 |
| 2010 | 215 | 46.85298 | -102.206 | RR + | JPL | 10 |
| 2010 | 216 | 46.86258 | -102.058 | RR + | JPL | 10 |
| 2010 | 217 | 46.863 | -101.977 | RR + | JPL | 0.1 |
| 2010 | 218 | 46.8617 | -101.836 | RR + | JPL | 0.25 |
| 2010 | 219 | 46.86157 | -101.719 | Null | JPL | 0.05 |
| 2010 | 220 | 46.86148 | -101.589 | none | JPL | 0 |
| 2010 | 221 | 46.86123 | -101.479 | RR + | JPL | 0.1 |
| 2010 | 222 | 46.86093 | -101.351 | none | JPL | 0 |
| 2010 | 223 | 46.86203 | -101.247 | RR + | JPL | 0.02 |
| 2010 | 224 | 46.8621 | -101.138 | none | JPL | 0 |
| 2010 | 225 | 46.86268 | -101.031 | none | JPL | 0 |
| 2010 | 226 | 46.84983 | -100.92 | RR + | JPL | 0.01 |
| 2010 | 227 | 46.83043 | -100.807 | none | JPL | 0 |
| 2010 | 228 | 47.18737 | -100.855 | none | JPL | 0 |
| 2010 | 229 | 47.22547 | -100.934 | none | JPL | 0 |
| 2010 | 230 | 47.2779 | -101.005 | none | JPL | 0 |
| 2010 | 231 | 47.30302 | -101.036 | RR + | JPL | 0.06 |
| 2010 | 232 | 47.26402 | -101.116 | RR + | JPL | 0.01 |
| 2010 | 233 | 47.2371 | -101.21 | LL+ | JPL | 0.03 |
| 2010 | 234 | 47.25692 | -101.3 | RR + | JPL | 0.01 |
| 2010 | 235 | 47.29473 | -101.381 | none | JPL | 0 |
| 2010 | 236 | 47.29927 | -101.488 | RR + | JPL | 0.01 |
| 2010 | 237 | 47.29937 | -101.588 | RR + | JPL | 0.77 |
| 2010 | 238 | 47.29922 | -101.686 | LL+ | JPL | 0.04 |
| 2010 | 239 | 47.30277 | -101.786 | LL+ | JPL | 0.08 |
| 2010 | 240 | 47.29212 | -101.887 | none | JPL | 0 |
| 2010 | 241 | 47.30147 | -101.981 | none | JPL | 0 |
| 2010 | 242 | 47.2992 | -102.073 | none | JPL | 0 |
| 2010 | 243 | 47.29962 | -102.176 | none | JPL | 0 |
| 2010 | 244 | 47.31377 | -102.274 | Null | JPL | 0.02 |
| 2010 | 245 | 47.31512 | -102.334 | RR + | JPL | 0.06 |
| 2010 | 246 | 47.34277 | -102.415 | none | JPL | 0 |
| 2010 | 247 | 47.34367 | -102.514 | none | JPL | 0 |
| 2010 | 248 | 47.34958 | -102.616 | RR + | JPL | 0.01 |
| 2010 | 249 | 47.3575 | -102.706 | none | JPL | 0 |
| 2010 | 250 | 47.35768 | -102.816 | none | JPL | 0 |
| 2010 | 251 | 47.35773 | -102.923 | none | JPL | 0 |
| 2010 | 252 | 47.35738 | -103.031 | none | JPL | 0 |
| 2010 | 253 | 47.34325 | -103.128 | none | JPL | 0 |
| 2010 | 254 | 47.36073 | -103.202 | none | JPL | 0 |
| 2010 | 255 | 47.42722 | -103.248 | none | JPL | 0 |
| 2010 | 256 | 47.49865 | -103.248 | none | JPL | 0 |
| 2010 | 257 | 47.57078 | -103.238 | none | JPL | 0 |
| 2010 | 258 | 47.62825 | -103.27 | none | JPL | 0 |
| 2010 | 259 | 47.69925 | -103.283 | none | JPL | 0 |
| 2010 | 260 | 47.7557 | -103.283 | none | JPL | 0 |
| 2010 | 261 | 47.79903 | -103.314 | none | JPL | 0 |
| 2010 | 262 | 47.80488 | -103.42 | none | JPL | 0 |
| 2010 | 263 | 47.8461 | -103.642 | none | JPL | 0 |
| 2010 | 264 | 47.86272 | -104 | none | JPL | 0 |
| 2010 | 265 | 48.13998 | -103.722 | none | JPL | 0 |
| 2010 | 266 | 48.14717 | -103.602 | LL+ | JPL | 0.01 |
| 2010 | 267 | 48.15438 | -103.485 | none | JPL | 0 |
| 2010 | 268 | 48.15443 | -103.381 | none | JPL | 0 |
| 2010 | 269 | 48.15672 | -103.275 | none | JPL | 0 |
| 2010 | 270 | 48.17848 | -103.188 | none | JPL | 0 |
| 2010 | 271 | 48.1817 | -103.109 | none | JPL | 0 |
| 2010 | 272 | 48.1974 | -103.023 | none | JPL | 0 |
| 2010 | 273 | 48.19722 | -102.911 | none | JPL | 0 |
| 2010 | 274 | 48.20685 | -102.813 | none | JPL | 0 |
| 2010 | 275 | 48.21145 | -102.709 | none | JPL | 0 |
| 2010 | 276 | 48.20393 | -102.613 | none | JPL | 0 |
| 2010 | 277 | 48.13183 | -102.613 | none | JPL | 0 |
| 2010 | 278 | 48.05968 | -102.609 | none | JPL | 0 |
| 2010 | 279 | 48.0347 | -102.528 | none | JPL | 0 |
| 2010 | 280 | 47.97998 | -102.459 | none | JPL | 0 |
| 2010 | 281 | 47.97775 | -102.341 | none | JPL | 0 |
| 2010 | 282 | 47.97773 | -102.236 | RR + | JPL | 0.1 |
| 2010 | 283 | 47.97783 | -102.128 | RR + | JPL | 1 |
| 2010 | 284 | 47.97783 | -102.022 | RR + | JPL | 0.85 |
| 2010 | 285 | 47.97818 | -101.915 | Null | JPL | 0.37 |
| 2010 | 286 | 47.97787 | -101.815 | RR + | JPL | 0.38 |
| 2010 | 287 | 47.97807 | -101.708 | RR + | JPL | 0.2 |
| 2010 | 288 | 47.97817 | -101.605 | RR + | JPL | 0.07 |
| 2010 | 289 | 47.9787 | -101.504 | RR + | JPL | 0.11 |
| 2010 | 290 | 47.97865 | -101.401 | RR + | JPL | 0.02 |
| 2010 | 291 | 47.97882 | -101.292 | RR + | JPL | 0.04 |
| 2010 | 292 | 47.97887 | -101.192 | LL+ | JPL | 0.15 |
| 2010 | 293 | 47.97927 | -101.09 | LL+ | JPL | 2.08 |
| 2010 | 294 | 47.99307 | -100.984 | RR + | JPL | 2 |
| 2010 | 295 | 48.02342 | -100.929 | RR + | JPL | 10 |
| 2010 | 296 | 48.0553 | -100.931 | RR + | JPL | 2 |
| 2010 | 297 | 48.0205 | -100.856 | RR + | JPL | 2 |
| 2010 | 298 | 48.00623 | -100.736 | RR + | JPL | 0.16 |
| 2010 | 299 | 47.97823 | -100.634 | RR + | JPL | 0.16 |
| 2010 | 300 | 47.9502 | -100.538 | RR + | JPL | 0.02 |
| 2010 | 301 | 47.8883 | -100.521 | none | JPL | 0 |
| 2010 | 302 | 47.83325 | -100.549 | none | JPL | 0 |
| 2010 | 303 | 47.83345 | -100.65 | none | JPL | 0 |
| 2010 | 304 | 47.82658 | -100.76 | none | JPL | 0 |
| 2010 | 305 | 47.8339 | -100.859 | none | JPL | 0 |
| 2010 | 306 | 47.81925 | -100.961 | Null | JPL | 0.18 |
| 2010 | 307 | 47.8194 | -101.066 | none | JPL | 0 |
| 2010 | 308 | 47.81928 | -101.162 | Null | JPL | 0.02 |
| 2010 | 309 | 47.81935 | -101.268 | LL+ | JPL | 0.02 |
| 2010 | 310 | 47.83383 | -101.293 | LL+ | JPL | 0.17 |
| 2010 | 311 | 47.9226 | -101.291 | RR + | JPL | 0.06 |
| 2010 | 312 | 48.05598 | -101.296 | none | JPL | 0 |
| 2010 | 313 | 47.80932 | -101.293 | RR + | JPL | 0.75 |
| 2010 | 314 | 47.74657 | -101.293 | LL+ | JPL | 0.43 |
| 2010 | 315 | 47.67412 | -101.289 | LL+ | JPL | 1.07 |
| 2010 | 316 | 47.64693 | -101.37 | LL+ | JPL | 0.01 |
| 2010 | 317 | 47.63943 | -101.474 | none | JPL | 0 |
| 2010 | 318 | 47.64657 | -101.579 | none | JPL | 0 |
| 2010 | 319 | 47.64648 | -101.686 | none | JPL | 0 |
| 2010 | 320 | 47.64652 | -101.791 | LL+ | JPL | 0.01 |
| 2010 | 321 | 47.57443 | -101.26 | RR + | JPL | 0.06 |
| 2010 | 322 | 47.51623 | -101.185 | RR + | JPL | 0.02 |
| 2010 | 323 | 47.50205 | -101.287 | none | JPL | 0 |
| 2010 | 324 | 47.4583 | -101.124 | RR + | JPL | 0.11 |
| 2010 | 325 | 47.45838 | -101.005 | LL+ | JPL | 0.01 |
| 2010 | 326 | 47.46472 | -100.908 | LL+ | JPL | 0.01 |
| 2010 | 327 | 47.48717 | -100.837 | none | JPL | 0 |
| 2010 | 328 | 47.48722 | -100.732 | none | JPL | 0 |
| 2010 | 329 | 47.46915 | -100.63 | RR + | JPL | 0.01 |
| 2010 | 330 | 47.47662 | -100.527 | LL+ | JPL | 0.07 |
| 2010 | 331 | 47.48727 | -100.413 | Null | JPL | 0.01 |
| 2010 | 332 | 47.48695 | -100.305 | RR + | JPL | 0.08 |
| 2010 | 333 | 47.48567 | -100.19 | RR + | JPL | 0.03 |
| 2010 | 334 | 47.46032 | -100.119 | LL+ | JPL | 0.02 |
| 2010 | 335 | 47.44323 | -100.033 | none | JPL | 0 |
| 2010 | 336 | 47.44317 | -99.9263 | RR + | JPL | 0.44 |
| 2010 | 337 | 47.44312 | -99.8196 | none | JPL | 0 |
| 2010 | 338 | 47.45765 | -99.7222 | none | JPL | 0 |
| 2010 | 339 | 47.4577 | -99.6186 | Null | JPL | 0.07 |
| 2010 | 340 | 47.45757 | -99.5116 | RR + | JPL | 0.01 |
| 2010 | 341 | 47.45737 | -99.4022 | Null | JPL | 0.05 |
| 2010 | 342 | 47.45763 | -99.293 | LL+ | JPL | 0.18 |
| 2010 | 343 | 47.45748 | -99.1952 | Null | JPL | 0.08 |
| 2010 | 344 | 47.45645 | -99.1319 | RR + | JPL | 0.1 |
| 2010 | 345 | 47.45707 | -98.9947 | RR + | JPL | 0.02 |
| 2010 | 346 | 47.457 | -98.8834 | LL+ | JPL | 0.03 |
| 2010 | 347 | 47.45703 | -98.7805 | Null | JPL | 0.02 |
| 2010 | 348 | 47.45715 | -98.6767 | none | JPL | 0 |
| 2010 | 349 | 47.45687 | -98.5757 | LL+ | JPL | 0.02 |
| 2010 | 350 | 47.44237 | -98.4738 | none | JPL | 0 |
| 2010 | 351 | 47.4424 | -98.3357 | none | JPL | 0 |
| 2010 | 352 | 47.44237 | -98.2135 | none | JPL | 0 |
| 2010 | 353 | 47.44188 | -98.1093 | RR + | JPL | 0.05 |
| 2010 | 354 | 47.43847 | -97.9959 | none | JPL | 0 |
| 2010 | 355 | 47.44173 | -97.8832 | none | JPL | 0 |
| 2010 | 356 | 47.47805 | -97.836 | RR + | JPL | 0.01 |
| 2010 | 357 | 47.51393 | -97.7829 | RR + | JPL | 0.02 |
| 2010 | 358 | 47.5207 | -97.6806 | none | JPL | 0 |
| 2010 | 359 | 47.52745 | -97.5757 | none | JPL | 0 |
| 2010 | 360 | 47.52715 | -97.4839 | none | JPL | 0 |
| 2010 | 361 | 47.49828 | -97.3939 | none | JPL | 0 |
| 2010 | 362 | 47.49787 | -97.2698 | LL+ | JPL | 0.04 |
| 2010 | 363 | 47.49775 | -97.1827 | none | JPL | 0 |
| 2010 | 364 | 47.48013 | -97.0767 | Null | JPL | 0.1 |
| 2010 | 365 | 47.40012 | -97.074 | none | JPL | 0 |
| 2010 | 366 | 47.35328 | -97.0515 | LL+ | JPL | 0.05 |
| 2010 | 367 | 47.28183 | -97.0202 | RR + | JPL | 0.03 |
| 2010 | 368 | 47.19353 | -96.9903 | RR + | JPL | 0.05 |
| 2010 | 369 | 47.11668 | -96.959 | Null | JPL | 0.03 |
| 2010 | 370 | 47.04993 | -96.9307 | RR + | JPL | 0.08 |
| 2010 | 372 | 46.63027 | -96.8346 | Null | JPL | 0.07 |
| 2010 | 373 | 46.63023 | -96.9432 | LL+ | JPL | 0.24 |
| 2010 | 374 | 46.62995 | -97.0557 | Null | JPL | 0.05 |
| 2010 | 375 | 46.62968 | -97.1657 | LL+ | JPL | 0.02 |
| 2010 | 376 | 46.6294 | -97.2757 | RR + | JPL | 1.7 |
| 2010 | 377 | 46.62932 | -97.3851 | RR + | JPL | 0.71 |
| 2010 | 378 | 47.77345 | -97.1105 | Null | MS | 0.05 |
| 2010 | 379 | 47.77375 | -97.2202 | none | MS | 0 |
| 2010 | 380 | 47.7591 | -97.3033 | RR + | MS | 0.01 |
| 2010 | 381 | 47.7447 | -97.3776 | none | MS | 0 |
| 2010 | 382 | 47.74443 | -97.4741 | LL+ | MS | 0.27 |
| 2010 | 383 | 47.74445 | -97.5629 | LL+ | MS | 0.37 |
| 2010 | 384 | 47.74477 | -97.6602 | Null | MS | 0.02 |
| 2010 | 385 | 47.7447 | -97.7642 | none | MS | 0 |
| 2010 | 386 | 47.74477 | -97.8607 | LL+ ; RR+ | MS | 0.01 |
| 2010 | 387 | 47.74472 | -97.9578 | none | MS | 0 |
| 2010 | 388 | 47.74507 | -98.0508 | none | MS | 0 |
| 2010 | 389 | 47.74937 | -98.1506 | LL+ | MS | 0.02 |
| 2010 | 390 | 47.78827 | -98.2794 | none | MS | 0 |
| 2010 | 391 | 47.80278 | -98.3764 | none | MS | 0 |
| 2010 | 392 | 47.80283 | -98.4831 | none | MS | 0 |
| 2010 | 393 | 47.80012 | -98.5867 | none | MS | 0 |
| 2010 | 394 | 47.7241 | -98.5901 | none | MS | 0 |
| 2010 | 395 | 47.68702 | -98.6606 | none | MS | 0 |
| 2010 | 396 | 47.68707 | -98.7478 | LL+ | MS | 0.02 |
| 2010 | 397 | 47.68723 | -98.8554 | none | MS | 0 |
| 2010 | 398 | 47.68727 | -98.9648 | none | MS | 0 |
| 2010 | 399 | 47.68735 | -99.0723 | none | MS | 0 |
| 2010 | 400 | 47.646 | -99.1326 | none | MS | 0 |
| 2010 | 401 | 47.64565 | -99.181 | LL+ | MS | 0.02 |
| 2010 | 402 | 47.64558 | -99.2857 | none | MS | 0 |
| 2010 | 403 | 47.64597 | -99.3785 | Null | MS | 0.05 |
| 2010 | 404 | 47.64578 | -99.4798 | none | MS | 0 |
| 2010 | 405 | 47.64625 | -99.5861 | none | MS | 0 |
| 2010 | 406 | 47.68593 | -99.7263 | Null | MS | 0.03 |
| 2010 | 407 | 47.71678 | -99.8099 | none | MS | 0 |
| 2010 | 408 | 47.746 | -99.8929 | RR + | MS | 0.01 |
| 2010 | 409 | 47.78707 | -99.9781 | LL+ | MS | 0.02 |
| 2010 | 410 | 47.81595 | -100.079 | RR + | MS | 0.09 |
| 2010 | 411 | 47.85377 | -100.177 | RR + | MS | 0.02 |
| 2010 | 412 | 47.89078 | -100.267 | LL+ | MS | 0.03 |
| 2010 | 413 | 47.92018 | -100.377 | Null | MS | 0.81 |
| 2010 | 414 | 48.63077 | -98.8083 | LL+ | MS | 0.09 |
| 2010 | 415 | 48.63135 | -98.9156 | LL+ | MS | 0.28 |
| 2010 | 416 | 48.63122 | -98.0316 | LL+ | MS | 0.02 |
| 2010 | 417 | 48.63115 | -99.1338 | LL+ | MS | 0.08 |
| 2010 | 418 | 48.63148 | -99.2423 | Null | MS | 0.91 |
| 2010 | 419 | 48.63178 | -99.3578 | Null | MS | 0.04 |
| 2010 | 420 | 48.632 | -99.4715 | LL+ | MS | 0.08 |
| 2010 | 421 | 48.63198 | -99.5806 | Null | MS | 0.05 |
| 2010 | 422 | 48.66097 | -99.667 | LL+ | MS | 0.07 |
| 2010 | 423 | 48.6609 | -99.7719 | RR + | MS | 0.15 |
| 2010 | 424 | 48.67547 | -99.8735 | LL+ | MS | 0.02 |
| 2010 | 425 | 48.67698 | -99.972 | LL+ | MS | 0.06 |
| 2010 | 426 | 48.63058 | -98.6922 | LL+ | MS | 0.34 |
| 2010 | 427 | 48.63032 | -98.5792 | LL+ | MS | 0.08 |
| 2010 | 428 | 48.6302 | -98.4705 | LL+ | MS | 0.05 |
| 2010 | 429 | 48.63052 | -98.3664 | LL+ | MS | 0.02 |
| 2010 | 430 | 48.63035 | -98.2749 | LL+ | MS | 3.06 |
| 2010 | 431 | 48.62995 | -98.1663 | LL+ | MS | 0.16 |
| 2010 | 432 | 48.63018 | -98.0643 | Null | MS | 0.05 |
| 2010 | 433 | 48.62993 | -98.9709 | LL+ | MS | 0.06 |
| 2010 | 434 | 48.62282 | -97.8624 | RR + | MS | 0.04 |
| 2010 | 435 | 48.6008 | -97.7967 | LL+ | MS | 0.17 |
| 2010 | 436 | 48.60237 | -97.6936 | Null | MS | 0.04 |
| 2010 | 437 | 48.60105 | -97.596 | none | MS | 0 |
| 2010 | 438 | 48.6005 | -97.4908 | none | MS | 0 |
| 2010 | 439 | 48.57172 | -97.4256 | Null | MS | 0.31 |
| 2010 | 440 | 48.57165 | -97.3215 | LL+ | MS | 0.02 |
| 2010 | 441 | 48.57115 | -97.2123 | none | MS | 0 |
| 2010 | 442 | 48.76102 | -98.2557 | none | MS | 0 |
| 2010 | 443 | 48.7613 | -98.1573 | LL+ | MS | 0.11 |
| 2010 | 444 | 48.761 | -98.054 | LL+ | MS | 0.48 |
| 2010 | 445 | 48.76102 | -97.9346 | RR + | MS | 0.14 |
| 2010 | 446 | 48.76075 | -97.8401 | Null | MS | 0.04 |
| 2010 | 447 | 48.77308 | -97.734 | LL+ | MS | 0.16 |
| 2010 | 448 | 48.79342 | -97.6281 | none | MS | 0 |
| 2010 | 449 | 48.80443 | -97.5236 | LL+ | MS | 0.05 |
| 2010 | 450 | 48.804 | -97.4143 | LL+ | MS | 0.14 |
| 2010 | 451 | 48.80323 | -97.2939 | RR + | MS | 0.04 |
| 2010 | 452 | 48.8038 | -97.2132 | none | MS | 0 |
| 2010 | 453 | 48.414 | -98.8402 | none | MS | 0 |
| 2010 | 454 | 48.41352 | -98.7315 | none | MS | 0 |
| 2010 | 455 | 48.41305 | -98.6229 | LL+ | MS | 0.02 |
| 2010 | 456 | 48.41285 | -98.5141 | none | MS | 0 |
| 2010 | 457 | 48.41293 | -98.4054 | none | MS | 0 |
| 2010 | 458 | 48.41307 | -98.3026 | LL+ | MS | 0.03 |
| 2010 | 459 | 48.41272 | -98.1879 | LL+ | MS | 0.02 |
| 2010 | 460 | 48.41242 | -98.0792 | LL+ | MS | 0.01 |
| 2010 | 461 | 48.487 | -98.9777 | LL+ | MS | 0.01 |
| 2010 | 462 | 48.39768 | -97.862 | none | MS | 0 |
| 2010 | 463 | 48.39855 | -97.7584 | LL+ | MS | 0.07 |
| 2010 | 464 | 48.39805 | -97.6481 | LL+ | MS | 0.08 |
| 2010 | 465 | 48.41228 | -97.5574 | Null | MS | 0.1 |
| 2010 | 466 | 48.41222 | -97.4484 | none | MS | 0 |
| 2010 | 467 | 48.41233 | -97.349 | none | MS | 0 |
| 2010 | 468 | 48.41198 | -97.2448 | Null | MS | 0.09 |
| 2010 | 469 | 48.45182 | -98.8843 | LL+ | MS | 0.21 |
| 2010 | 470 | 48.48628 | -98.9228 | none | MS | 0 |
| 2010 | 471 | 48.412 | -98.0142 | none | MS | 0 |
| 2010 | 472 | 48.4865 | -99.1139 | Null | MS | 0.04 |
| 2010 | 473 | 48.48677 | -99.2039 | Null | MS | 0.02 |
| 2010 | 474 | 48.48685 | -99.3191 | Null | MS | 0.02 |
| 2010 | 475 | 48.48693 | -99.437 | Null | MS | 0.01 |
| 2010 | 476 | 48.49518 | -99.5264 | none | MS | 0 |
| 2010 | 477 | 48.5018 | -99.6237 | none | MS | 0 |
| 2010 | 478 | 48.5016 | -99.7286 | RR + | MS | 0.12 |
| 2010 | 479 | 48.50128 | -99.8198 | none | MS | 0 |
| 2010 | 480 | 48.50262 | -99.9065 | RR + | MS | 0.08 |
| 2010 | 481 | 48.50167 | -99.9883 | none | MS | 0 |
| 2010 | 482 | 48.50175 | -100.081 | none | MS | 0 |
| 2010 | 483 | 48.50402 | -100.175 | none | MS | 0 |
| 2010 | 484 | 48.55062 | -100.229 | none | MS | 0 |
| 2010 | 485 | 48.59217 | -100.277 | none | MS | 0 |
| 2010 | 486 | 48.619 | -100.299 | none | MS | 0 |
| 2010 | 487 | 48.33862 | -100.403 | none | MS | 0 |
| 2010 | 488 | 48.35458 | -100.346 | none | MS | 0 |
| 2010 | 489 | 48.35495 | -100.274 | LL+ | MS | 0.08 |
| 2010 | 490 | 48.35468 | -100.172 | Null | MS | 0.43 |
| 2010 | 491 | 48.35477 | -100.069 | Null | MS | 0.21 |
| 2010 | 492 | 48.35445 | -99.9652 | Null | MS | 0.07 |
| 2010 | 493 | 48.35388 | -99.8691 | none | MS | 0 |
| 2010 | 494 | 48.33977 | -99.7901 | none | MS | 0 |
| 2010 | 495 | 48.33922 | -99.6966 | Null | MS | 0.02 |
| 2010 | 496 | 48.32452 | -99.6206 | none | MS | 0 |
| 2010 | 497 | 48.30702 | -99.5374 | RR + | MS | 0.07 |
| 2010 | 498 | 48.29178 | -99.4593 | Null | MS | 0.26 |
| 2010 | 499 | 48.28037 | -99.357 | RR + | MS | 0.13 |
| 2010 | 500 | 48.27653 | -99.2652 | LL+ | MS | 0.04 |
| 2010 | 501 | 48.26017 | -99.1791 | none | MS | 0 |
| 2010 | 502 | 48.2223 | -99.0992 | none | MS | 0 |
| 2010 | 503 | 48.14603 | -98.9329 | none | MS | 0 |
| 2010 | 504 | 48.1039 | -98.8686 | none | MS | 0 |
| 2010 | 505 | 48.76125 | -98.3917 | LL+ | MS | 15 |
| 2010 | 506 | 48.76157 | -98.4747 | RR + | MS | 20 |
| 2010 | 507 | 48.76152 | -98.5624 | LL+ | MS | 30 |
| 2010 | 508 | 48.76148 | -98.6499 | RR + | MS | 15 |
| 2010 | 509 | 48.7615 | -98.7373 | RR + | MS | 10 |
| 2010 | 510 | 48.76133 | -98.8368 | LL+ | MS | 15 |
| 2010 | 511 | 48.7905 | -98.9016 | Null | MS | 0.04 |
| 2010 | 512 | 48.79062 | -99.0007 | LL+ | MS | 0.08 |
| 2010 | 513 | 48.79038 | -99.0992 | LL+ | MS | 0.07 |
| 2010 | 514 | 48.79053 | -99.1983 | LL+ | MS | 0.37 |
| 2010 | 515 | 48.8052 | -99.2742 | LL+ | MS | 0.22 |
| 2010 | 516 | 48.85307 | -99.3508 | RR + | MS | 0.08 |
| 2010 | 517 | 48.8631 | -99.4609 | LL+ | MS | 0.3 |
| 2010 | 518 | 48.86303 | -99.5629 | LL+ | MS | 0.82 |
| 2010 | 519 | 48.84917 | -99.6571 | LL+ | MS | 0.01 |
| 2010 | 520 | 48.838 | -99.7489 | Null | MS | 0.02 |
| 2010 | 521 | 48.80853 | -99.8409 | RR + | MS | 0.36 |
| 2010 | 522 | 48.80368 | -99.9421 | Null | MS | 0.11 |
| 2010 | 523 | 48.80607 | -100.038 | LL+ | MS | 0.04 |
| 2010 | 524 | 48.80635 | -100.139 | Null | MS | 0.06 |
| 2010 | 525 | 48.8064 | -100.249 | RR + | MS | 0.04 |
| 2010 | 526 | 48.80855 | -100.358 | Null | MS | 0.12 |
| 2010 | 527 | 48.82087 | -100.451 | RR + | MS | 0.02 |
| 2010 | 528 | 48.82103 | -100.567 | LL+ | MS | 0.09 |
| 2010 | 529 | 48.82103 | -100.674 | RR + | MS | 0.04 |
| 2010 | 530 | 48.82103 | -100.786 | none | MS | 0 |
| 2010 | 531 | 48.82095 | -100.888 | none | MS | 0 |
| 2010 | 532 | 48.82122 | -101.001 | RR + | MS | 0.06 |
| 2010 | 533 | 48.82113 | -101.097 | none | MS | 0 |
| 2010 | 534 | 48.78845 | -101.147 | RR + | MS | 0.05 |
| 2010 | 535 | 48.76295 | -101.218 | LL+ | MS | 0.08 |
| 2010 | 536 | 48.22895 | -101.233 | none | MS | 0 |
| 2010 | 537 | 48.23925 | -101.112 | none | MS | 0 |
| 2010 | 538 | 48.23963 | -101.004 | LL+ | MS | 0.04 |
| 2010 | 539 | 48.23958 | -100.904 | RR + | MS | 0.08 |
| 2010 | 540 | 48.27347 | -100.815 | RR + | MS | 0.07 |
| 2010 | 541 | 48.2898 | -100.719 | none | MS | 0 |
| 2010 | 542 | 48.30711 | -100.617 | Null | MS | 0.01 |
| 2010 | 543 | 48.31165 | -100.518 | LL+ | MS | 0.02 |
| 2010 | 544 | 48.31163 | -100.42 | LL+ | MS | 0.01 |
| 2010 | 545 | 48.23368 | -101.377 | RR + | MS | 0.11 |
| 2010 | 546 | 48.282 | -101.435 | RR + | MS | 5.3 |
| 2010 | 547 | 48.3086 | -101.525 | LL+ | MS | 0.69 |
| 2010 | 548 | 48.31303 | -101.64 | none | MS | 0 |
| 2010 | 549 | 48.32753 | -101.748 | LL+ | MS | 0.03 |
| 2010 | 550 | 48.32742 | -101.86 | none | MS | 0 |
| 2010 | 551 | 48.32735 | -101.965 | none | MS | 0 |
| 2010 | 552 | 48.32578 | -102.078 | none | MS | 0 |
| 2010 | 553 | 48.32742 | -102.181 | none | MS | 0 |
| 2010 | 554 | 48.32708 | -102.289 | none | MS | 0 |
| 2010 | 555 | 48.30745 | -102.393 | RR + | MS | 0.19 |
| 2010 | 556 | 48.31317 | -102.505 | RR + | MS | 0.05 |
| 2010 | 557 | 48.31308 | -102.606 | RR + | MS | 0.05 |
| 2010 | 558 | 48.31527 | -102.723 | none | MS | 0 |
| 2010 | 559 | 48.3422 | -102.828 | RR + | MS | 0.06 |
| 2010 | 560 | 48.34263 | -102.937 | none | MS | 0 |
| 2010 | 561 | 48.34298 | -103.044 | RR + | MS | 0.01 |
| 2010 | 562 | 48.34263 | -103.147 | LL+ | MS | 0.01 |
| 2010 | 563 | 48.3425 | -103.249 | none | MS | 0 |
| 2010 | 564 | 48.34267 | -103.356 | none | MS | 0 |
| 2010 | 565 | 48.3429 | -103.475 | none | MS | 0 |
| 2010 | 566 | 48.34257 | -103.583 | none | MS | 0 |
| 2010 | 567 | 48.29422 | -103.626 | none | MS | 0 |
| 2010 | 568 | 48.22665 | -103.626 | none | MS | 0 |
| 2010 | 569 | 48.16492 | -103.638 | none | MS | 0 |
| 2010 | 570 | 48.14052 | -103.734 | none | MS | 0 |
| 2010 | 571 | 48.14042 | -103.839 | none | MS | 0 |
| 2010 | 572 | 48.3158 | -101.516 | RR + | MS | 0.07 |
| 2010 | 573 | 48.37235 | -101.579 | LL+ | MS | 0.05 |
| 2010 | 574 | 48.41568 | -101.654 | LL+ | MS | 0.14 |
| 2010 | 575 | 48.4608 | -101.743 | RR + | MS | 0.19 |
| 2010 | 576 | 48.48782 | -101.84 | LL+ | MS | 0.11 |
| 2010 | 577 | 48.53828 | -101.933 | LL+ | MS | 0.24 |
| 2010 | 578 | 48.54652 | -102.023 | Null | MS | 0.11 |
| 2010 | 579 | 48.54638 | -102.131 | RR + | MS | 0.01 |
| 2010 | 580 | 48.5464 | -102.241 | LL+ | MS | 0.01 |
| 2010 | 581 | 48.54728 | -102.356 | LL+ | MS | 0.02 |
| 2010 | 582 | 48.56087 | -102.441 | none | MS | 0 |
| 2010 | 583 | 48.56107 | -102.56 | RR + | MS | 0.1 |
| 2010 | 584 | 48.57512 | -102.655 | none | MS | 0 |
| 2010 | 585 | 48.57533 | -102.766 | none | MS | 0 |
| 2010 | 586 | 48.57547 | -102.865 | none | MS | 0 |
| 2010 | 587 | 48.59 | -102.962 | none | MS | 0 |
| 2010 | 588 | 48.59003 | -103.071 | none | MS | 0 |
| 2010 | 589 | 48.57557 | -103.168 | none | MS | 0 |
| 2010 | 590 | 48.57552 | -103.277 | none | MS | 0 |
| 2010 | 591 | 48.58112 | -103.386 | none | MS | 0 |
| 2010 | 592 | 48.57578 | -103.494 | none | MS | 0 |
| 2010 | 593 | 48.57092 | -103.603 | none | MS | 0 |
| 2010 | 594 | 48.573 | -103.711 | none | MS | 0 |
| 2010 | 595 | 48.7633 | -101.321 | LL+ | MS | 0.09 |
| 2010 | 596 | 48.76318 | -101.436 | LL+ | MS | 0.05 |
| 2010 | 597 | 48.7636 | -101.541 | none | MS | 0 |
| 2010 | 598 | 48.7636 | -101.659 | LL+ | MS | 0.14 |
| 2010 | 599 | 48.7636 | -101.783 | LL+ | MS | 0.01 |
| 2010 | 600 | 48.76372 | -101.884 | LL+ | MS | 0.01 |
| 2010 | 601 | 48.76378 | -101.996 | LL+ | MS | 0.04 |
| 2010 | 602 | 48.77827 | -102.087 | RR + | MS | 0.02 |
| 2010 | 603 | 48.80735 | -102.161 | none | MS | 0 |
| 2010 | 604 | 48.82652 | -102.24 | LL+ | MS | 0.31 |
| 2010 | 605 | 48.8943 | -102.257 | Null | MS | 0.01 |
| 2010 | 606 | 48.89443 | -102.363 | RR + | MS | 0.06 |
| 2010 | 607 | 48.89445 | -102.475 | RR + | MS | 0.05 |
| 2010 | 608 | 48.89447 | -102.584 | none | MS | 0 |
| 2010 | 609 | 48.8944 | -102.699 | none | MS | 0 |
| 2010 | 610 | 48.89463 | -102.808 | Null | MS | 0.17 |
| 2010 | 611 | 48.89465 | -102.918 | none | MS | 0 |
| 2010 | 612 | 48.89452 | -103.034 | LL+ | MS | 0.16 |
| 2010 | 613 | 48.89453 | -103.137 | LL+ | MS | 0.01 |
| 2010 | 614 | 48.8947 | -103.246 | Null | MS | 0.01 |
| 2010 | 615 | 48.909 | -103.356 | none | MS | 0 |
| 2010 | 616 | 48.90892 | -103.461 | none | MS | 0 |
| 2010 | 617 | 48.90902 | -103.575 | none | MS | 0 |
| 2010 | 618 | 48.90928 | -103.685 | none | MS | 0 |
| 2010 | 619 | 48.90882 | -103.8 | RR + | MS | 0.03 |
| 2010 | 620 | 48.06395 | -98.7841 | none | MS | 0 |
| 2010 | 621 | 48.0498 | -98.6815 | none | MS | 0 |
| 2010 | 622 | 48.04973 | -98.5934 | Null | MS | 0.02 |
| 2010 | 623 | 48.03532 | -98.454 | LL+ | MS | 0.09 |
| 2010 | 624 | 48.03532 | -98.3524 | none | MS | 0 |
| 2010 | 625 | 48.02785 | -98.2337 | none | MS | 0 |
| 2010 | 626 | 48.0209 | -98.1394 | LL+ | MS | 0.05 |
| 2010 | 627 | 48.01773 | -98.0321 | LL+ | MS | 0.01 |
| 2010 | 628 | 48.0052 | -97.9318 | Null | MS | 0.04 |
| 2010 | 629 | 48.00108 | -97.823 | none | MS | 0 |
| 2010 | 630 | 47.96228 | -97.7621 | Null | MS | 0.05 |
| 2010 | 631 | 47.94685 | -97.6453 | none | MS | 0 |
| 2010 | 632 | 47.93942 | -97.5349 | none | MS | 0 |
| 2010 | 633 | 47.93272 | -97.4362 | RR + | MS | 0.03 |
| 2010 | 634 | 47.93298 | -97.3267 | RR + | MS | 0.02 |
